# Open-source three-dimensional IoT anemometer for indoor air quality monitoring

**DOI:** 10.1016/j.ohx.2025.e00656

**Published:** 2025-05-31

**Authors:** Elizabeth Ospina-Rojas, Juan Botero-Valencia, Daniel Betancur-Vasquez, Joshua M. Pearce

**Affiliations:** a Faculty of Engineering, Instituto Tecnológico Metropolitano, Grupo SCR, Medellín 050034, Colombia; b Faculty of Engineering, Institución Universitaria de Envigado, Grupo GITESI, Envigado 055420, Colombia; c Department of Electrical and Computer Engineering, Western University, Ontario N6A 3K7, Canada

**Keywords:** Three-dimensional anemometer, Thermal anemometer, Wind speed, Wind direction, IoT sensors, Indoor air quality, Open hardware

## Abstract

Ventilation in an enclosed space can significantly influence people’s comfort, health, and safety. Poor ventilation can generate temperatures dangerous to health or obstruct the dispersion of environmental pollutants, such as toxic gases or pollution. Measuring indoor environmental conditions can thus help improve the quality of the environment and protect people’s health and comfort. This work proposes the design of an open-source anemometer to measure wind speed and direction in three dimensions. The purpose of this anemometer is to monitor wind conditions in enclosed spaces and environmental conditions related to air quality and temperature. The prototype uses an array of six unidirectional flow sensors, each pointing towards a different axis. Carbon dioxide (CO${}_{2}$), volatile organic compounds (VOC), temperature, humidity, pressure, and gas presence sensors are integrated to monitor indoor environmental conditions accurately. Measuring the vertical component of the wind provides more detailed information on wind conditions. Test results show that the device can detect variations in wind speed with a deviation of 0.25 m/s, detect changes in horizontal wind direction with a deviation of 3.7°, and detect vertical wind direction variations with a deviation of 3.02°. These measurements demonstrate that the proposed device is capable of detecting wind changes in three dimensions, validating its potential for detailed indoor airflow monitoring.

## Graphical abstract

**Figure:**
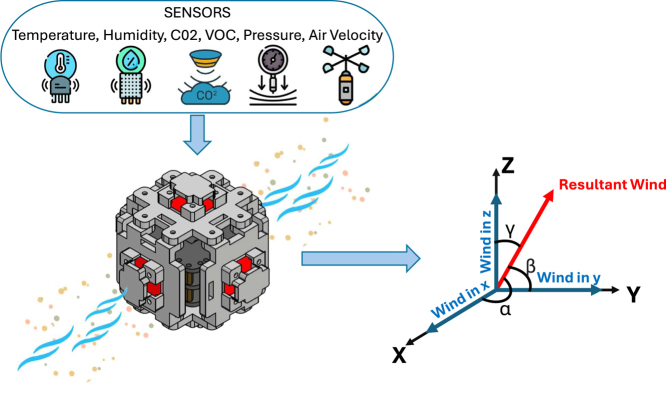

## Specifications table

**Table:** 

Hardware name	Three-dimensional anemometer for indoor air quality monitoring
Subject area	•Engineering •Instrumentation •Anemometry •Air quality monitoring •Internet of things

Hardware type	•Measuring physical properties and in-lab sensors •Field measurements and sensors •Electrical engineering and computer science

Open source license	Creative Commons Attribution-ShareAlike license
Cost of hardware	$522.26
Source file repository	https://doi.org/10.17605/OSF.IO/MZCTP

## 1 Hardware in context

Wind is a vector quantity characterized by speed and direction, typically obtained in two dimensions and, and in some cases three dimensions [1]. This variable is a fundamental factor in measurements for climate and meteorological analysis, scientific, medical and industrial applications, autonomous maritime vehicles, wind power generation, building construction or indoor air quality control, among others [2], [3], [4], [5], [6], [7].

The meteorological instrument implemented to characterize the wind is the anemometer, which is responsible for measuring the wind speed at a given point and, in some cases, the direction. Different types of anemometers are named according to their configuration, the most popular are mechanical anemometers, ultrasonic anemometers, and thermal anemometers [8].

Mechanical anemometers are based on a system of cups and a rotor that rotates when the wind pushes the system, in this way the speed of rotation is related to the wind speed. The limitations of these anemometers are the over speed, mechanical limitations, and changes in the measurement due to extreme weather conditions [9], [10], [11]. Ultrasonic anemometers provide measurements not only for wind speed, but also for determining the predominant direction of airflow [12] either in two or three dimensions and promise to be more accurate than mechanical anemometers, however, they may have limitations for outdoor use and the measurement may be affected by the angle of attack of the wind [13], in addition, they are highly sensitive to noise [14], [15], [16].

Thermal anemometers operate by utilizing a heater that is cooled by the surrounding environment. The temperature variations detected in the heater can be correlated with the velocity of the air that cools it. These anemometers find widespread use in indoor wind monitoring applications owing to their compact size. Additionally, they are employed in the detection of various gases. The high sensitivity of these anemometers enables the measurement of very low air velocities, although it is worth noting that they are not suitable for outdoor use [8].

In recent years, the use of sensors based on microelectromechanical systems (MEMS) to measure different environmental variables such as wind has increased. MEMS used to measure wind speed have the advantage of low power consumption and compact size. Thermal MEMS operate by quantifying heat transfer between an element and the surrounding fluid. Piezoresistive MEMS detect changes in material resistance caused by air currents, while piezoelectric MEMS serve as elements converting mechanical energy into electrical energy and vice versa [17].

Anemometers are used in different areas where wind plays a fundamental role, their area of application depends on the configuration of the anemometer, whether it is mechanical or electronic. Mechanical anemometers are mainly used in outdoor environments to monitor weather conditions in open spaces, they are used as an aid to generate predictions about the weather or future storms and are used in wind power generation farms to obtain as much energy as possible from air currents, and they are also widely used to monitor wind conditions to control transportation systems such as air, rail, or maritime since wind conditions affect the correct operation of these systems [18], [19], [20].

In agriculture, anemometers play a crucial role in monitoring wind conditions. This monitoring is essential to understand the influence of wind on pest growth and disease spread [21]. Additionally, anemometers facilitate the precise application of fertilizers and pesticides using unmanned agricultural helicopters [22]. In the work by Garcia [23], an electronic anemometer is utilized to analyze the air discharge of a sprayer commonly used for distributing pesticides in crop fields. This study demonstrates that anemometers are also instrumental in analyzing the development of machines and tools that operate with air [24]. This approach contributes to more efficient and targeted dosing, optimizing agricultural practices, and promoting sustainable resource management. In addition, they are used in weather stations for solar photovoltaic systems and agrivoltaics (the combination of agriculture and photovoltaic) [25].

Unlike mechanical anemometers, electronic anemometers are mainly used indoors or in enclosed spaces. This is because their sensors are often sensitive and fragile when exposed to humidity or adverse outdoor conditions. A significant portion of electronic anemometers are based on thermal sensors, which relate airspeed to the cooling of a heater. Their use is therefore restricted to environments with low humidity, as moisture can cause measurement errors or directly damage the device’s electronics. Electronic anemometers are essential for analyzing and calibrating ventilation or air expulsion systems. Their ability to measure lower air velocities surpasses that of mechanical anemometers, making them particularly suitable for tasks that require smooth airflow or high accuracy.

In indoor spaces, anemometers are used to verify the performance of ventilation systems, air conditioning, and air quality. Ventilation in enclosed spaces can significantly impact people’s comfort, health, and safety. Poor ventilation can lead to dangerous temperatures or hinder the dissipation of environmental pollutants, such as toxic gases or airborne particles [26], [27]. Unfortunately, indoor air monitoring systems are not common due to the high cost and fragility of available sensors on the market [1], [28]. The ability to monitor indoor environmental conditions at a low cost provides an opportunity to improve the quality of the environment, protecting human health and comfort.

The development of low-cost three-dimensional sensors for measuring environmental variables is a relevant area of research, even more so if technology is articulated that can be deployed in sensory networks [12], [20]. These types of technologies feed Internet of Things (IoT) networks for the creation of massive datasets since they can be implemented on a large scale [19]. Measuring environmental variables in three dimensions with an electronic system is a challenge for the scientific community and converges on implementations of a high level of knowledge of physics [29], so hardware for low-cost sensor networks has been the subject of research in recent academic studies [25], [30], [31].

IoT environmental sensors are being widely used in various areas. For example, in [32], the authors use IoT sensors for temperature, humidity, and TDS to remotely monitor the performance and water quality of a desalination system for producing drinking water. In [33], a system is developed for the early detection of forest fires, utilizing IoT sensors for humidity, temperature, carbon dioxide, and rain presence. This system sends remote alerts to a monitoring center upon detecting a fire, allowing timely decisions to prevent its spread.

In [34], the environmental conditions of an indoor kale crop are monitored using an IoT platform with UV light, humidity, air, and soil sensors, ensuring the necessary conditions for plant growth. Continuing with IoT sensor applications in agriculture, [35] implements a crop data collection system where general environmental variables, volatile organic compounds (VOC) presence, and carbon dioxide levels are measured. The collected data are used to predict climatic conditions and forecast unfavorable conditions for crop growth.

Various configurations for indoor environmental condition monitoring systems can be found. In [36], an IoT device is developed for detecting VOC, gases, and odors produced by different types of mosquito repellents used indoors. Similarly, in [37], a system is implemented to measure VOC, CO, CO${}_{2}$ concentrations, humidity, and temperature in indoor spaces. The data are sent to the cloud using IoT technology and visualized in a web application; the device is equipped with lights that change hue according to pollution levels in the environment. In [38], IoT sensors are used to measure temperature, humidity, pressure, CO, CO${}_{2}$, and PM2.5. The collected data are then used to train an air quality prediction model, focusing on indoor air quality analysis.

Open-source projects encourage the transfer of knowledge among experts [39]. By making a development public, developers around the world can contribute to its improvement, correct bugs, make updates, and propose new uses and applications [40], which increases the scalability of the projects [41]. By freely disclosing the development of a prototype, different users can access this information and, based on it, create their developments without having to start from scratch, which reduces manufacturing times and associated costs [42].

In addition, open-source projects are often developed using technologies that are accessible and compatible with different systems, such as 3D prototyping and the use of Arduino-compatible programmable electronic devices [43]. On the Internet, there is a wide variety of repositories [44] where 3D designs that can be modified and adapted to the needs of each person can be downloaded for free [45]. A large amount of fully customizable code and software is also available.

The main advantage of open-source resources is that they allow developers to focus on innovation rather than on the technical aspects of development [46], which drives the creation of novel, high-value products, reducing manufacturing times and costs.

This work proposes a novel open-source anemometer with integrated environmental sensors for indoor air quality monitoring. The physical structure of the anemometer was 3D printed, and its compact and simple design facilitates its installation and adaptation to different environments, especially in enclosed spaces.

This anemometer is composed of six flow sensors that point to the six spatial coordinates, allowing to obtain wind measurements in three dimensions and to calculate the direction of the prevailing flow. In addition, it is equipped with sensors for carbon dioxide (CO${}_{2}$), volatile organic compounds (VOC), temperature, humidity, pressure, and presence of gases. All these variables can be used for air quality analysis, the detection of toxic or harmful agents for health, and the issuance of alerts.

## 2 Hardware description

**Fig. 1:**
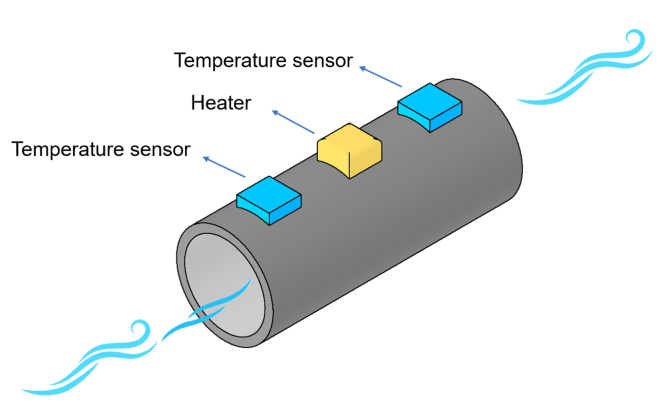
Basic hot-wire principle.

The hot wire principle is based on heating a thin metal film that is exposed to the environment, temperature sensors measure the progressive cooling of the metal film over some time. The rate at which the metal cools is related to the speed of the wind passing through it. Wind sensors based on the hot wire principle measure the temperature of the metal using thermopiles, which are electronic devices that deliver a voltage output depending on the temperature change detected in the metal film. In this way, the voltage output of the thermopiles can be related to the wind speed that cools the metal film. The better the resolution of the thermopiles, the more accurate the wind speed measurement. Fig. 1 illustrates the basic principle of the hot wire. A stream of air flows through a duct, cooling the metal heater inside. The temperature variation of the heater is measured by nearby sensors and is related to the wind speed.

This work proposes the design of a novel 3D thermal anemometer for measuring and monitoring indoor wind speed and direction. This anemometer utilizes microelectromechanical systems (MEMS) hot-wire wind sensors, known for their compact size and fast response times. Inside this prototype, environmental sensors were added to measure CO${}_{2}$, VOC, temperature, humidity, pressure, and gas presence sensors.

Traditional hot-wire anemometers only measure wind flow in one direction due to the principle behind their operation. To overcome this limitation and achieve the goal of three-dimensional wind measurement, this design employs an array of six strategically positioned hot-wire sensors. These sensors capture wind speed data along the three axes of the coordinate space (x, y, and z).

The six sensors are positioned perpendicular to each other and are labeled according to their measurement axis. Two sensors point to the positive and negative X-axis, two sensors point to the positive and negative Y-axis, and the last two to the positive and negative Z-axis, in this way the wind speed can be measured in three dimensions. The structure of this anemometer is similar to that of a cube, with a sensor on each face.

By acquiring wind speed information in all three spatial directions, basic mathematical techniques can be applied to calculate the resultant wind speed and direction as explained in Section 2.8. This approach enables comprehensive wind characterization within an indoor environment, providing valuable data for various applications.

The entire structure of the proposed anemometer was 3D-printed on a RepRap class fused filament fabrication-based 3-D printer (Creality K1 MAX 0.4 mm nozzle) [47], [48] using polylactic acid (PLA) material, parts of this anemometer are described in Section 3. See the configuration of the print parameters in Table 1.

**Fig. 2:**
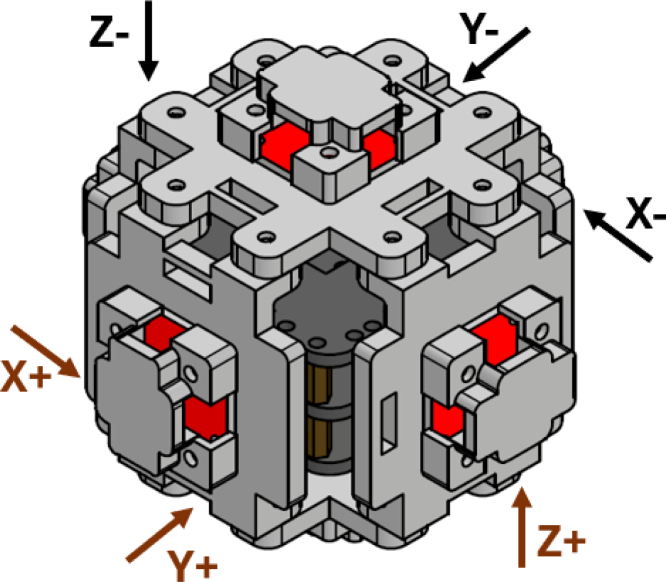
Labeled anemometer design.

The assembly has a top, a bottom, a front, and a side face. On the top and bottom faces are placed the sensors pointing towards the y-axis. On the front and back faces are the sensors pointing toward the z-axis, and on the left and right faces are the sensors pointing to the x-axis. Three sensors are pointing in the positive direction of the Cartesian plane and the other three are pointing in the negative direction of the Cartesian plane, all sensors form an angle of 90° to each other. Fig. 2 shows the general configuration of the proposed anemometer. The axis along which each sensor is oriented is labeled, with arrows indicating the direction of airflow through each sensor. The red arrows represent positive sensing, while the black arrows represent negative sensing.

**Table 1:** 3D printing settings.

Parameter	Configuration
Layer height	0.20 mm
Line width	0.42 mm
Infill density	15%
Infill pattern	Grid
Build plate adhesion	Brim
Printing temperature	220 ${}^{\circ }C$
Build plate temperature	30 ${}^{\circ }C$
Print speed	100 mm/s
Generate support	Check
Support structure	Normal
Support pattern	Grid
Support density	5%
Regular fan speed	70%

### 2.1 Sensors

**Fig. 3:**
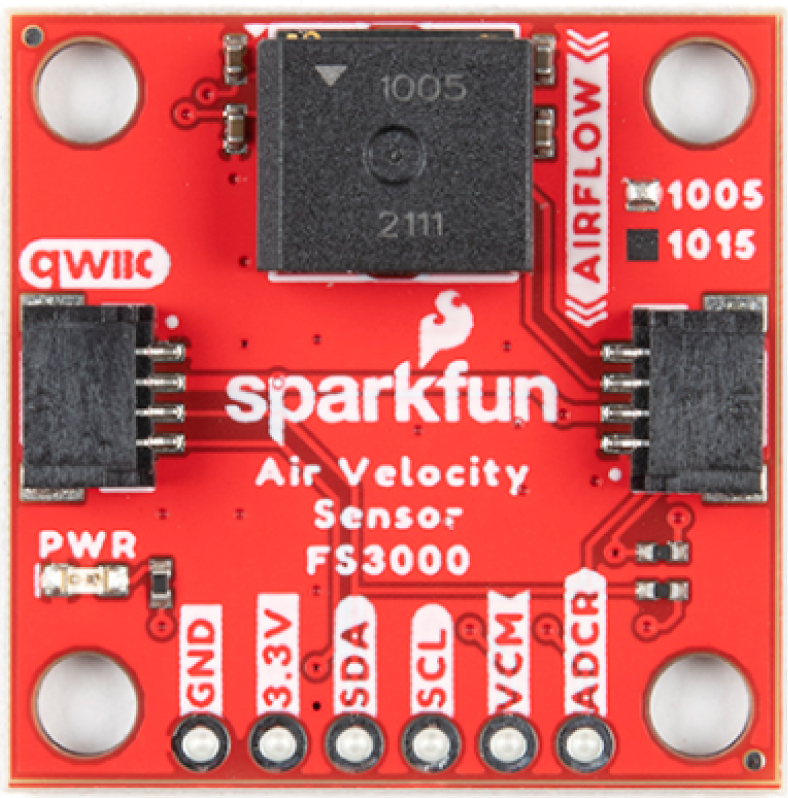
Flow sensor FS3000.

The FS3000 is an open-source module sourced from SparkFun Electronics (USA) based on the MEMS thermopile flow sensor FS3000-1005 that enables the measurement of air velocity in a single direction, see Fig. 3. This sensor works when an internal filament is heated and then cooled by the air circulating through the system. The thermopile is responsible for capturing these temperature variations in the filament. In this way, the air velocity can be related to the temperature change in the filament captured by the thermopile.

Data from this sensor can be read via I${}^{2}$C with a resolution of 12 bits. Table 2 summarizes important information about the FS3000 sensor. For additional information refer to [49].

**Fig. 4:**
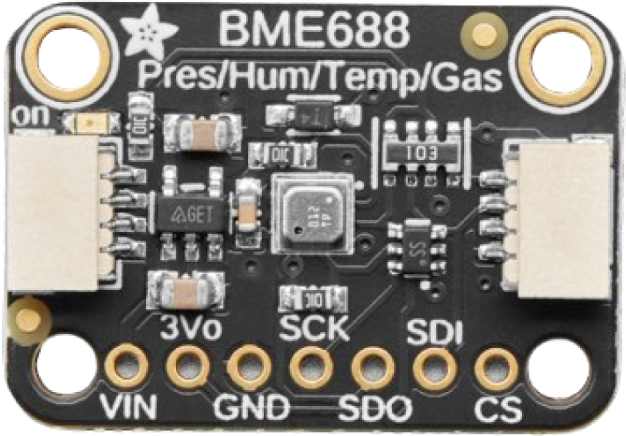
BME688 sensor.

**Fig. 5:**
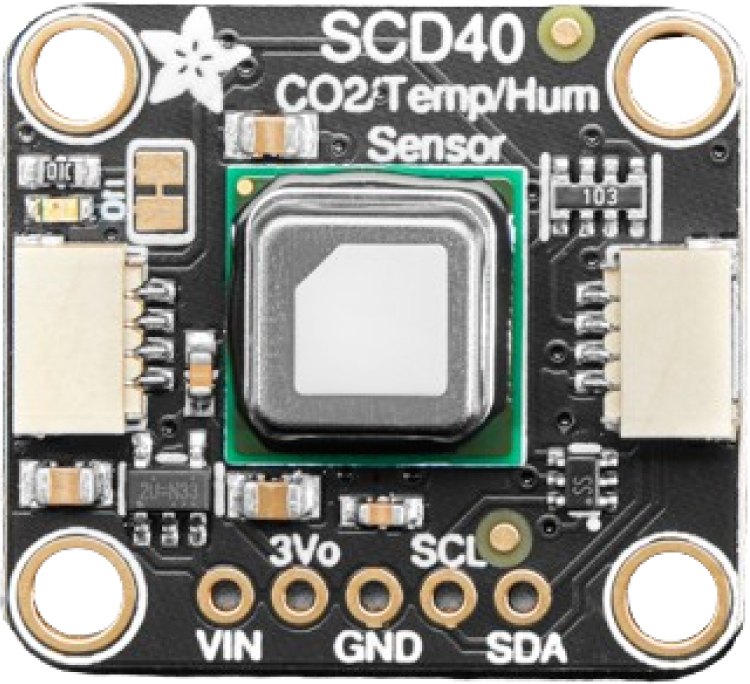
SCD40 sensor.

**Fig. 6:**
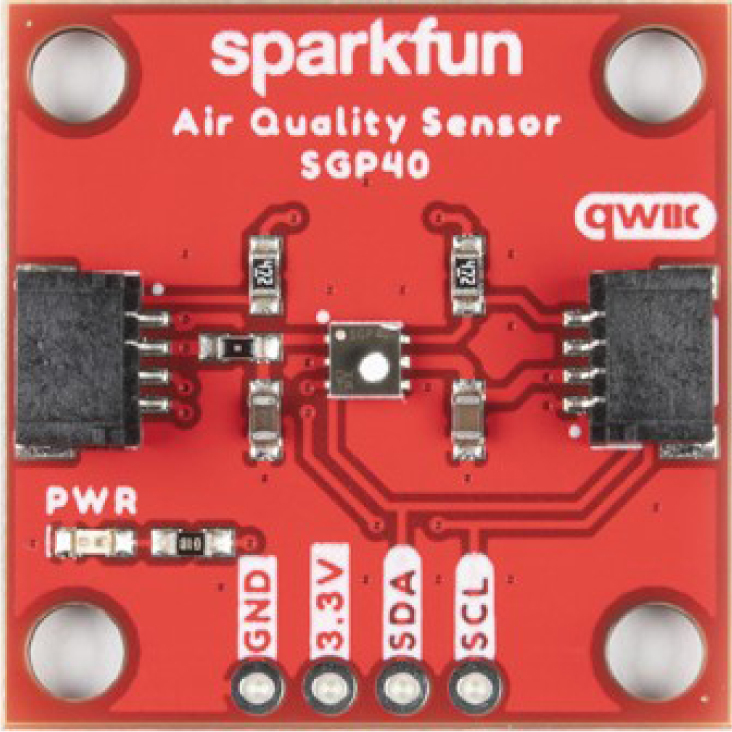
SGP40 sensor.

The open-source environmental sensors used in this project are the BME688 and SCD40 (Fig. 4, Fig. 5), sourced from AdaFruit (USA). Both sensors measure temperature and humidity. Additionally, the SCD40 measures CO${}_{2}$ concentrations in the environment. Furthermore, the SGP40 sensor, sourced from Sparkfun, provides measurements of VOC index related to air quality, Fig. 6. The data obtained from these sensors help to gain a deeper understanding of ambient conditions and air quality.

**Table 2:** The characteristics of the FS3000 sensor.

Characteristic	FS3000	Unit
Measurement range	0–7.23	[m/s]
Accuracy	5	[%]
Resolution	12	[bit]
Input Voltage	2.7–3.3	[V]
Average current draw	10	[mA]

### 2.2 Processor

The development board used to capture and process the sensor data is the open-source Sparkfun ESP32 Thing Plus (Fig. 8) which is based on the Tensilica Xtensa LX6 32-bit microprocessor and operates at a frequency of 240 MHz. The operating voltage of this board is between 2.3 V and 3.6 V and has an approximate power consumption of 80 mA which facilitates power supply. In addition, the WIFI module incorporated in this board allows the creation of IoT applications to send the wind data taken to the cloud and monitor them remotely.

### 2.3 Multiplexor

**Fig. 7:**
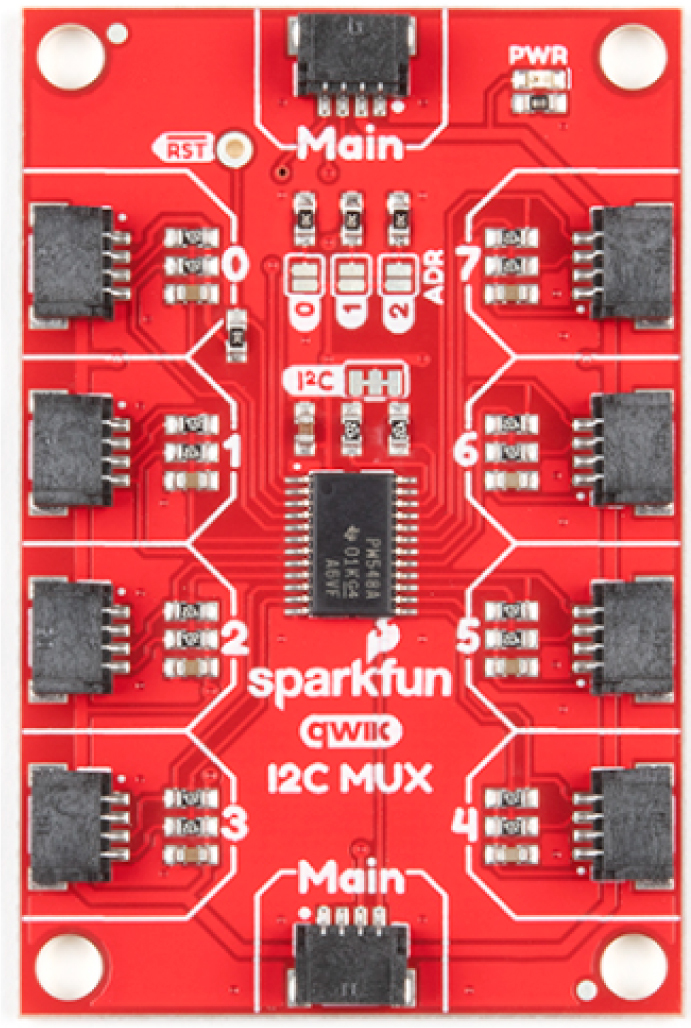
SparkFun Qwiic Mux Breakout (TCA9548A).

The FS3000 flow sensor has I${}^{2}$C communication which facilitates connections, however, its address cannot be changed. In this work, where a total of six of these sensors are used, a device is required to enable the selection of which sensor to use for measuring wind speed. The open source SparkFun Qwiic Mux Breakout (TCA9548A) facilitates the connection of up to 8 devices that share the same address (Fig. 7). The six FS3000 sensors are connected to this module so that the processor can select which one to measure from. This device has an operating voltage of 1.65 V–5.5 V and a maximum relay frequency of 400 kHz.

**Fig. 8:**
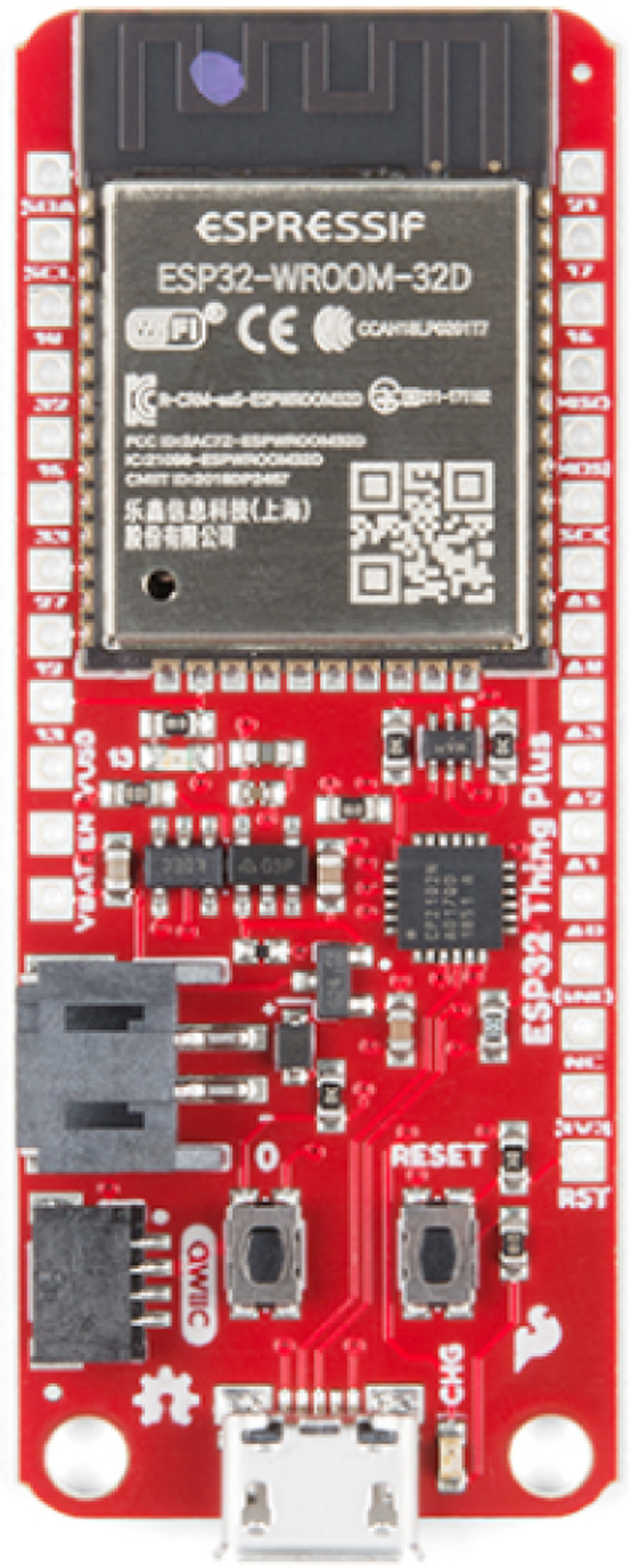
SparkFun Thing Plus - ESP32.

### 2.4 Data storage

**Fig. 9:**
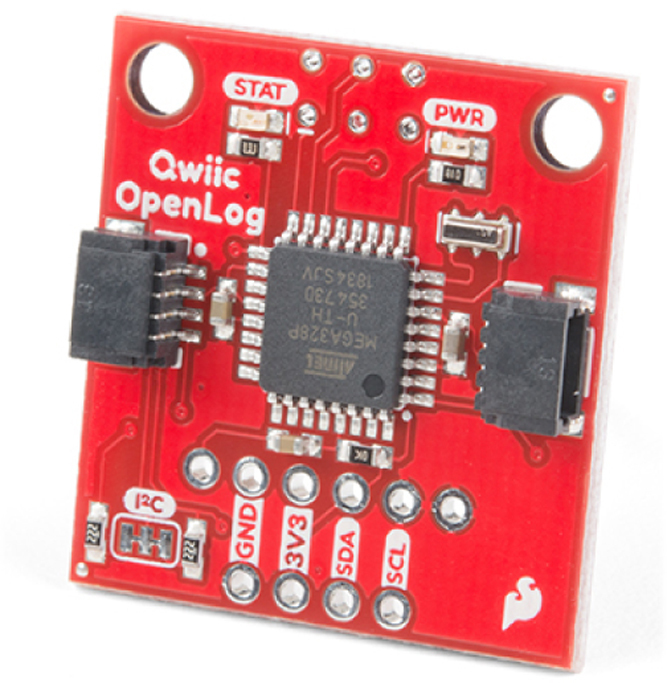
Sparkfun OpenLog data logger.

**Fig. 10:**
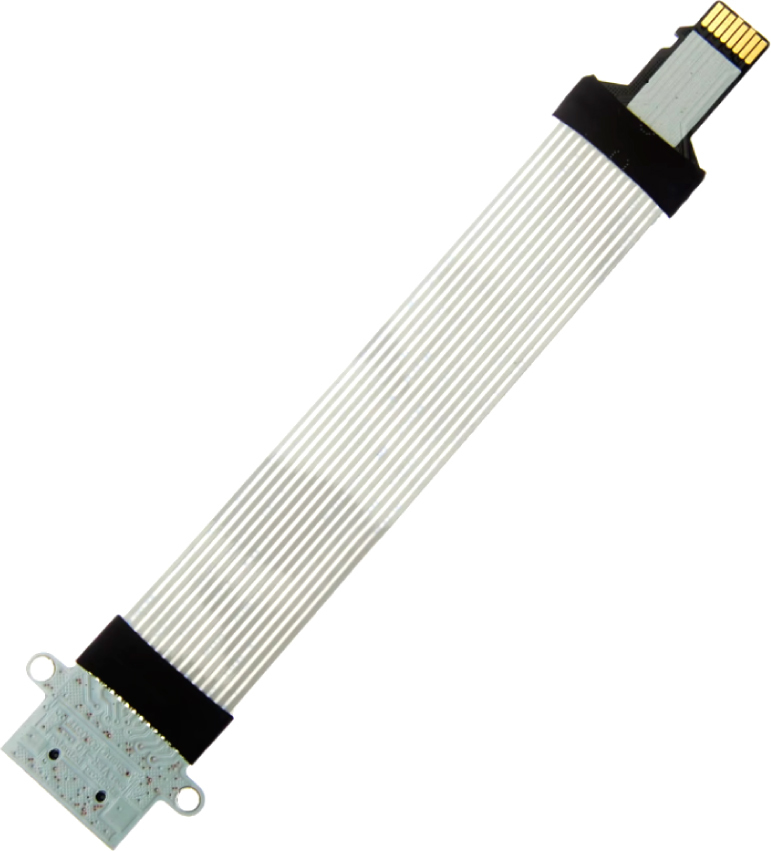
Micro SD memory extension cable.

The data taken from all the sensors are stored in a 16 GB micro SD memory, for this, the open source data logger in Fig. 9 is used, this device works with I${}^{2}$C communication that facilitates the connections. To facilitate the integration of OpenLog with the assembly and to locate the micro SD memory in a convenient and accessible position, we use an extension cable to connect the micro SD card, see Fig. 10.

### 2.5 Electrical design

**Fig. 11:**
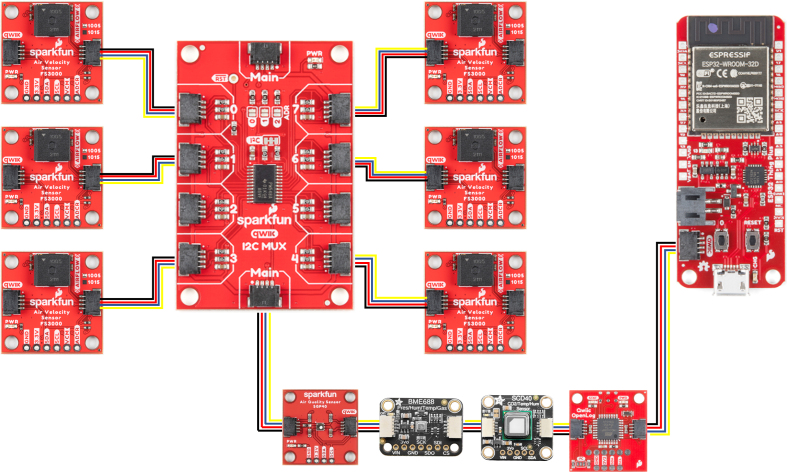
Electronic connection diagram.

Fig. 11 shows the electronic wiring diagram of the components used on this anemometer. To accommodate the use of six FS3000 sensors, which communicate via I${}^{2}$C and cannot have their addresses modified, the TCA9548A 1-to-8 I${}^{2}$C multiplexer was employed. Six of its outputs are used, with each sensor connected to a separate channel of the multiplexer. It is important to ensure that all selected channels operate at the same voltage as the sensors (3.3 V) to prevent errors. The SCD40, SGP40, and BME688 sensors are also connected to the multiplexer in series. Finally, the data logger is connected to the I${}^{2}$C bus of the microcontroller, specifically the SparkFun Thing Plus - ESP32. The sensors, multiplexer, data logger, and processor all feature Qwiic connectors, simplifying the connections.

### 2.6 Software

The ESP-32 is responsible for controlling the entire system. In the internal algorithm, the processor selects one of the six channels on the multiplexer where the six sensors are connected. Measurement is taken from one sensor at a time, first, the value of the positive x-axis is taken, and then the value of the negative x-axis, this process is repeated on the positive and negative y-axis sensors and the positive and negative z-axis sensors. All six values are stored on the device, When the reading from all sensors is stored, the processor calculates the resulting wind speed and the direction of the flow which is determined by the azimuth angle (horizontal) and the vertical angle, then the environmental variables of the BME688, SGP40, SCD40 sensors are measured. All data is stored on the micro SD card, with a separate .txt file created for each sensor. Each entry is taken each second, and includes the timestamp in Unix format.

The use of the ESP32 processor enables the integration of IoT technology, allowing data to be visualized and monitored remotely. The MQTT protocol can be used to transmit data from the device to various cloud platforms, such as Ubidots IoT Cloud, which facilitates the creation of interactive graphical environments for the user and simplifies the analysis of the data collected by the device. Fig. 13 illustrates how data can be sent to the cloud. The processor collects the data from the anemometer, performs the calculations and sends the data to the Ubidots platform by the MQTT protocol, for more detailed information see here.

**Fig. 12:**
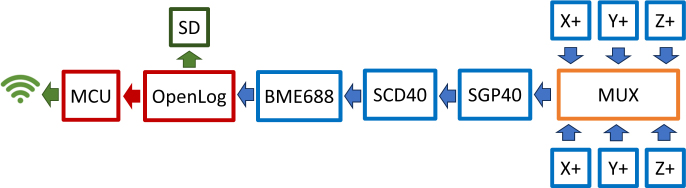
Block diagram of anemometer operation. (For interpretation of the references to color in this figure legend, the reader is referred to the web version of this article.)

Fig. 12 shows the general behavior of data collection and submission. The blue arrows represent the connections between the sensors, the multiplexer, and the data logger. The red arrow indicates the connection between the processor and the data logger. The processor selects the appropriate multiplexer channel, which retrieves the reading from the corresponding sensor and sends it back to the processor. Finally, the processed data is either stored on the micro SD card or sent to the cloud, as shown by the green arrow.

The software can be downloaded from this repo under the open-source GNU GPL v3 license.

The flowchart in Fig. 14 shows the general behavior of the implemented code. After the definition of the libraries, variables and sensors, the connection to Ubidots is configured, establishing the type of service, the token, the name and password of the WiFi network, as well as the time zone configuration for the NTP server.

**Fig. 13:**
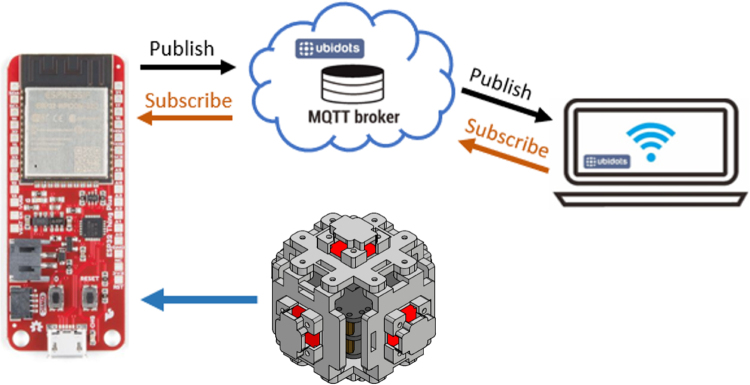
Data sending to the cloud.

Then, the connection of the sensors is verified individually, generating a reconnection loop in case any sensor is not detected. In addition, for each sensor, a message is issued in case it is not recognized. The connection to WiFi and Ubidots is established.

Then, the main loop is entered. Inside it, an additional loop is responsible for verifying the connection to Ubidots. This loop ensures that, if at any time the connection is interrupted, the device will attempt to reconnect and issue a corresponding message. If the connection is active, the process continues with sensor readings.

First, readings are taken from the FS3000 sensors, calculating wind speed and direction, and the data is stored in the microSD memory. Next, measurements are taken with the BME688 sensor and stored in the memory. The same process is repeated with the SCD40 and SGP40 sensors. At the end, all read data are sent in a single package to Ubidots.

**Fig. 14:**
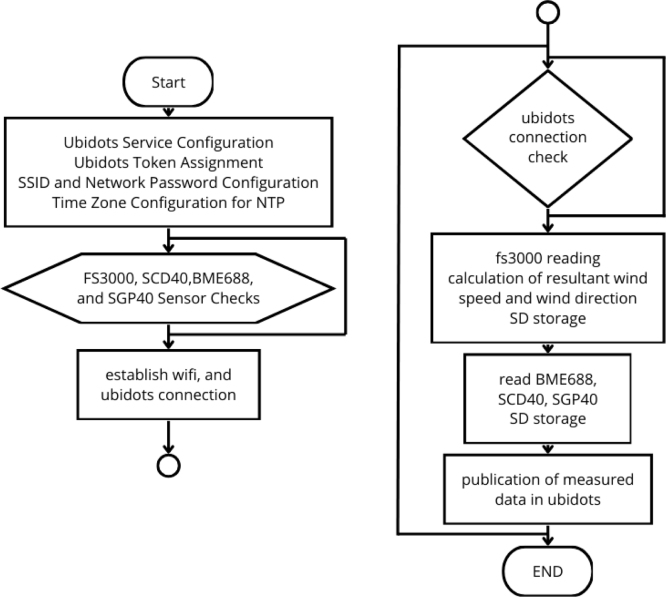
Algorithm flowchart.

### 2.7 IoT implementation

The integration of the ESP32 microcontroller enables the implementation of IoT technologies for transmitting and monitoring the data captured by the anemometer in the cloud. The ESP32 features a built-in WiFi module, which facilitates seamless and efficient internet connectivity.

In the device’s main code, the WiFi SSID and password are configured, allowing the system to automatically search for and establish a connection upon startup or reboot. Additionally, an NTP server is utilized to synchronize the local time, ensuring accurate timestamping of recorded data. Before proceeding with data acquisition, the system verifies that all sensors are properly connected and functioning.

Once connected to the WiFi network, the NTP server is configured with the appropriate time zone to obtain precise regional time. For cloud communication, dedicated libraries are employed to establish an MQTT connection. The Ubidots server (industrial.api.ubidots.com) is specified, and an authentication token, obtained from the personal Ubidots account, is defined.

To maintain a stable connection, a reconnection loop is implemented, ensuring that the device continuously attempts to reconnect in case of disconnection. Separate functions are defined to acquire data from the FS3000, SGP40, SCD40, and BME688 sensors. These functions are invoked within the main code, providing flexibility in sensor configuration. Each function generates a data string that is stored in a .txt file using the data logger, enabling local data backup on a microSD card.

Sensor readings are also formatted in JSON and published to the corresponding Ubidots topic. The data acquisition interval is set to five seconds to optimize power consumption, though this parameter can be adjusted based on application requirements. In indoor measurement scenarios, frequent updates may not be necessary, allowing for further optimization of resource usage.

### 2.8 Calculations

Since the flow sensors measure wind in only one direction, and taking advantage of the fact that wind speed is a vector involving multiple components, basic vector computation techniques were employed to determine both the magnitude and the direction of the resulting wind. In the processor, each of the wind speed components is stored individually: the wind speed on the positive and negative x-axis, the wind speed on the positive and negative y-axis, and the wind speed on the positive and negative z-axis. Once these measurements have been obtained, the resulting velocities for each axis are computed as shown in Eqs. (1) to (3).

(1) $$Vx=Vx_{pos}-Vx_{neg}$$

(2) $$Vy=Vy_{pos}-Vy_{neg}$$

(3) $$Vz=Vz_{pos}-Vz_{neg}$$

Finally, the resultant velocity Vw is calculated using the Eq. (4). In addition, to determine the direction of the wind the azimuth angle and the vertical angle are computed as is shown in Eqs. (5), (6).

(4) $$Vw=\sqrt{Vx^{2}+Vy^{2}+Vz^{2}}$$

(5) Azimuthal Angle=atan2(Vy,Vx)

(6) $$\text{vertical Angle}=\text{atan2}\left(Vz,\sqrt{Vx^{2}+Vy^{2}}\right)$$

## 3 Design files

**Fig. 15:**
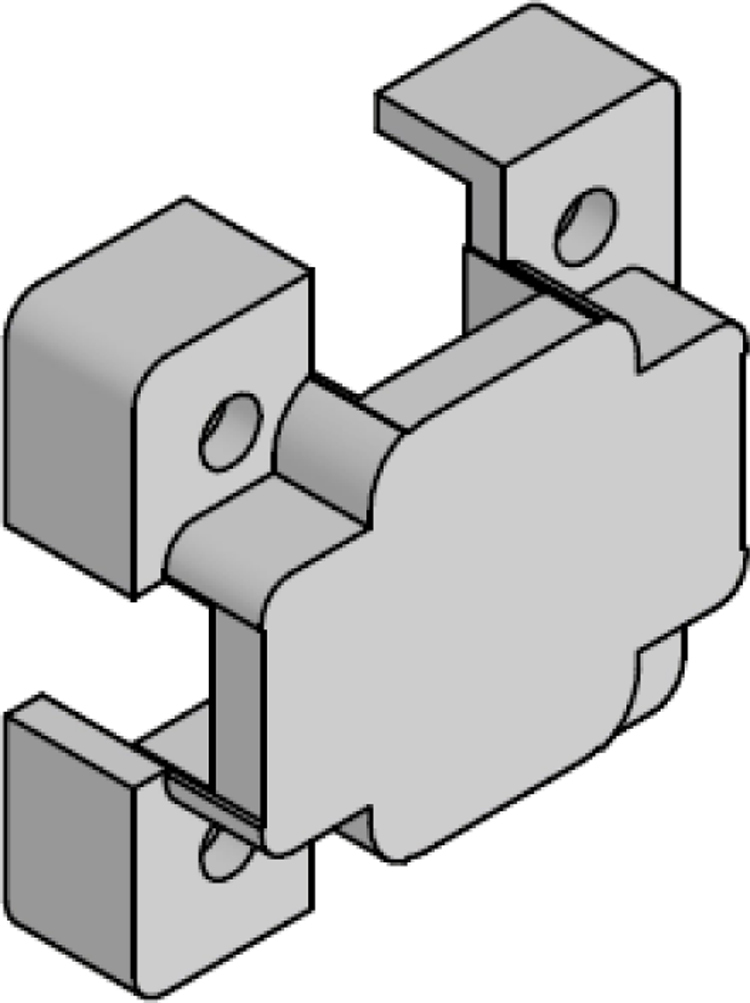
Sensor cover.

**Fig. 16:**
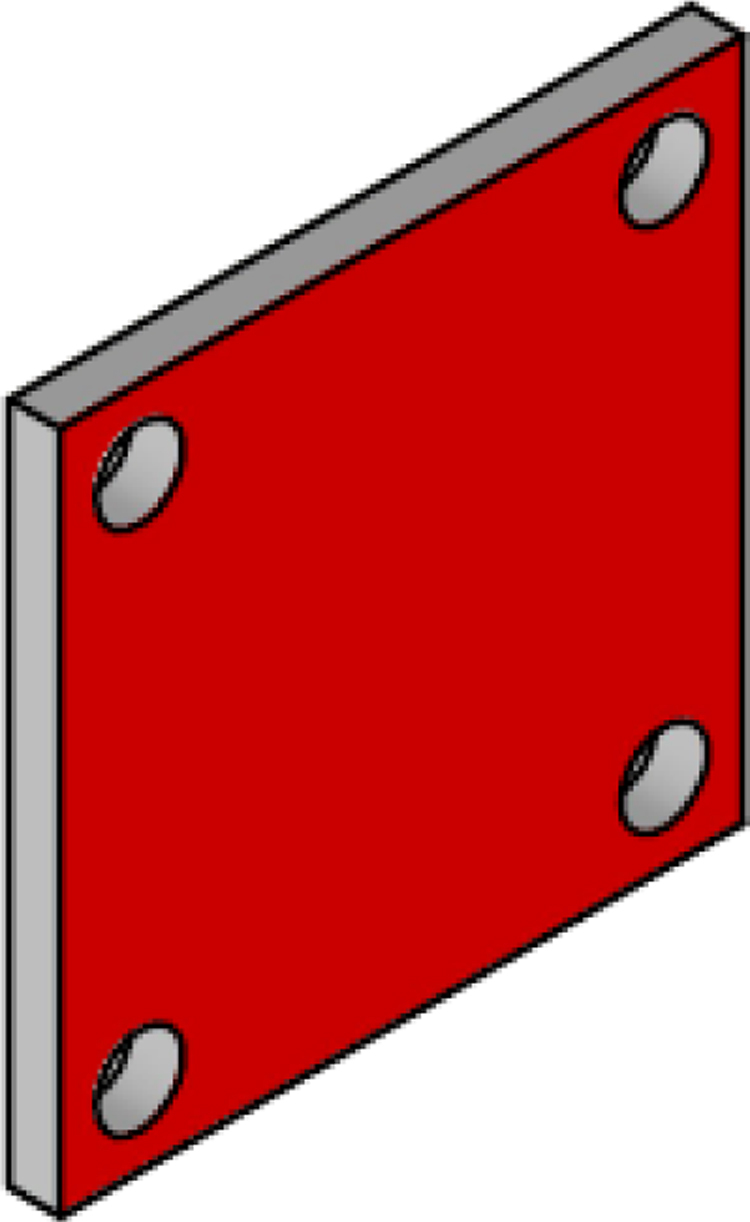
Sensor base.

The Fig. 24 illustrates the complete assembly with numbered parts. We begin by describing all the 3D-printed components. The part number 1, named sensor_cover (Fig. 15), was designed to enclose the FS3000 sensors’ electronics. Its primary purpose is to protect the sensors from environmental pollutants and other external agents that might interfere with the electronics, such as animals, insects, or mild humidity. The cover has lateral openings that allow airflow without compromising measurement accuracy, as well as cutouts to facilitate the passage of sensor cables. Part number 2, named sensor_base, is a 26.5 mm × 26.5 mm square with a thickness of 2 mm. This component serves as a platform for the fluid sensors, raising them slightly to improve fluid flow through the sensors, see Fig. 16.

**Fig. 17:**
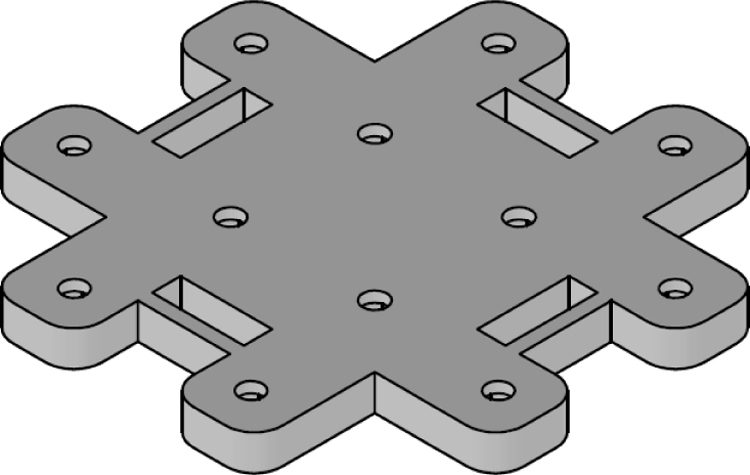
Y-axis sensor support.

Part number 3, Fig. 17, named y_axis_support, is used in pairs to mount the sensors oriented toward the positive and negative y-axis. Each piece measures 72 mm × 72 mm and includes four central holes to attach the sensor_base, where the flow sensor and sensor_cover are fixed. This component also has eight additional holes for securing the x_axis_support, z_axis_support, and z_axis_SD_support. Additionally, it features 13 mm × 4 mm slots for routing cables and can also be used to mount the anemometer to a surface, if needed.

**Fig. 18:**
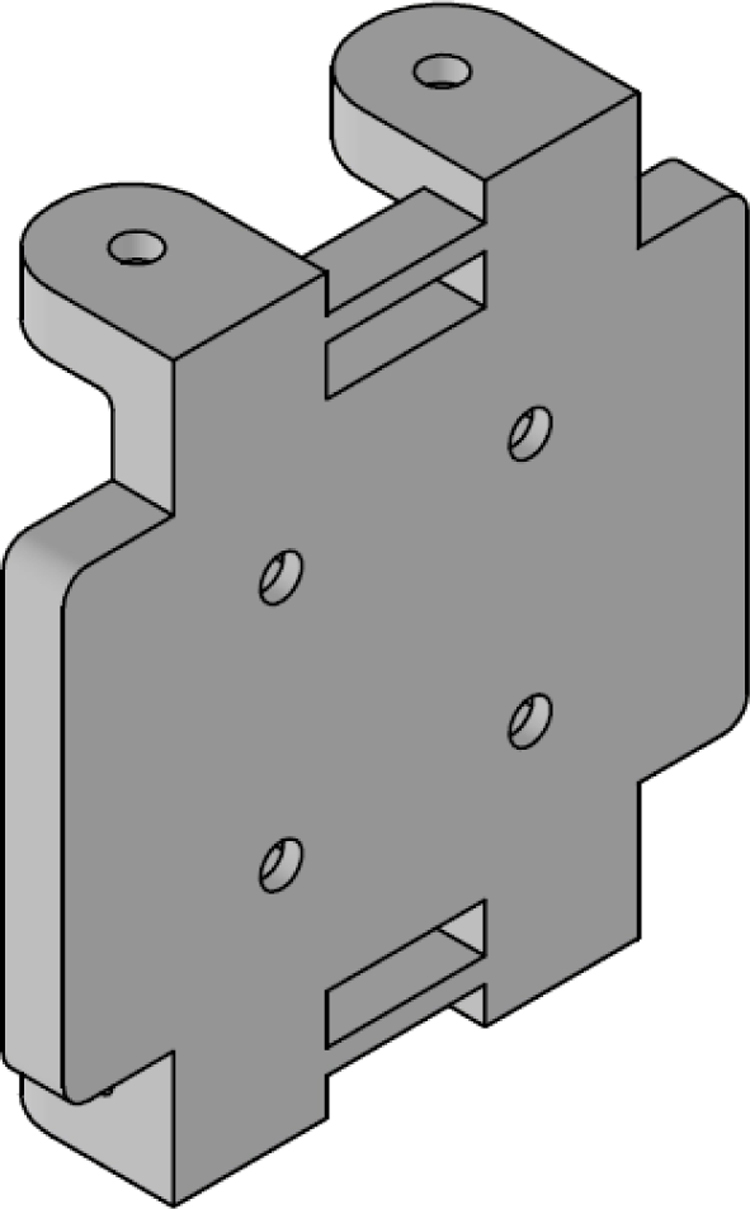
X-axis sensor.

The x_axis_support (part number 4) is designed to secure the x-positive and x-negative flow sensors, see Fig. 18. This part measures 60 mm × 56 mm and includes four holes for attaching the flow sensor, as well as four additional holes for connecting it to the upper and lower y_axis_support. It also features 13 mm × 4 mm slots for routing the sensor cables.

**Fig. 19:**
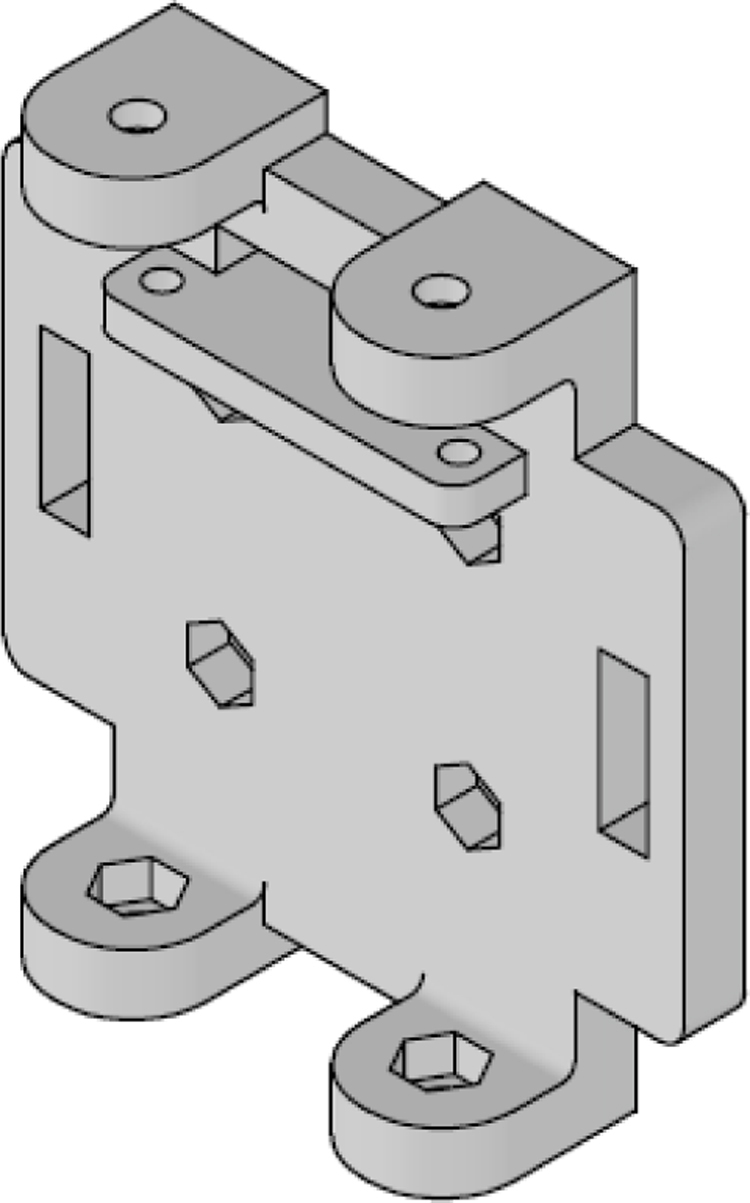
z-axis sensor with SD slot.

**Fig. 20:**
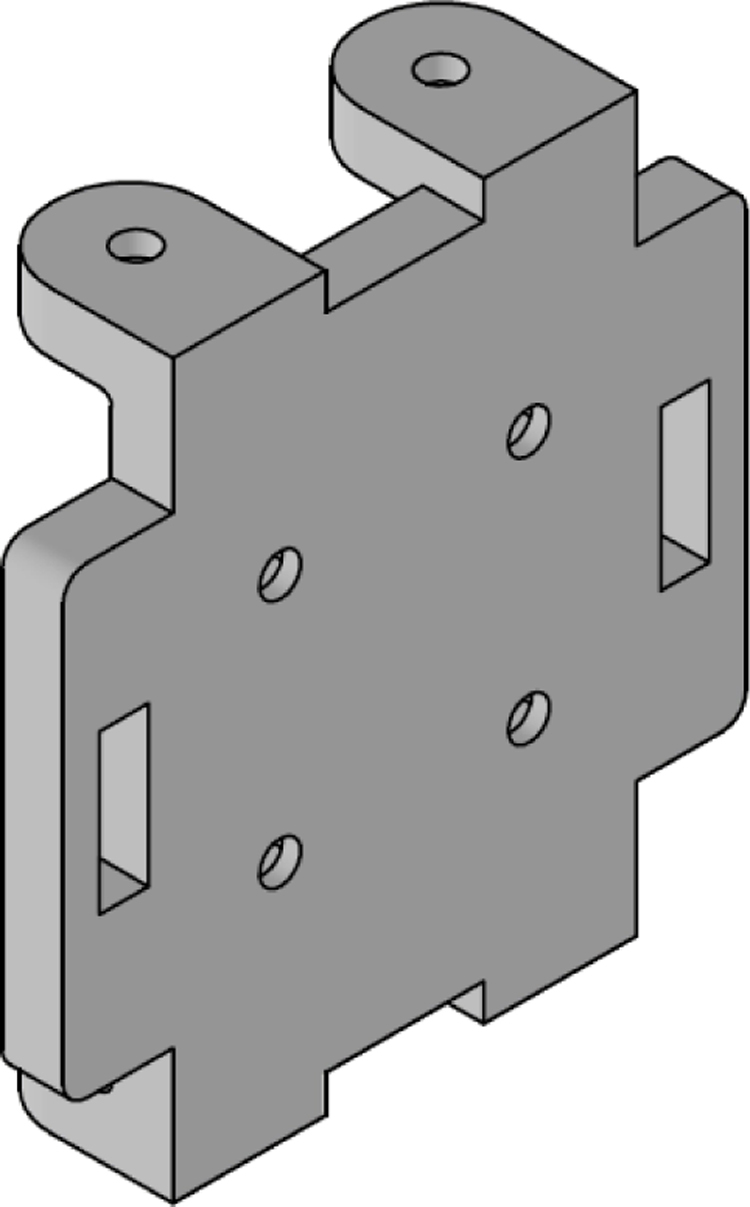
Z-axis sensor.

Parts 5 and 6, named z_axis_SD_support and z_axis_support, respectively, are used to mount the z-positive and z-negative flow sensors. In Fig. 19, Fig. 20 we want to show the 21 mm × 3.5 mm slot for the micro SD memory and the tab used to fix the extension cable which is fixed with M2 screws. This is the only feature that differentiates part 5 from part 6. All perforations in the pieces have a diameter of 3 mm, each piece has hexagonal holes on its back side to hold the nuts in place, preventing them from moving, it can be seen on Fig. 24.

**Fig. 21:**
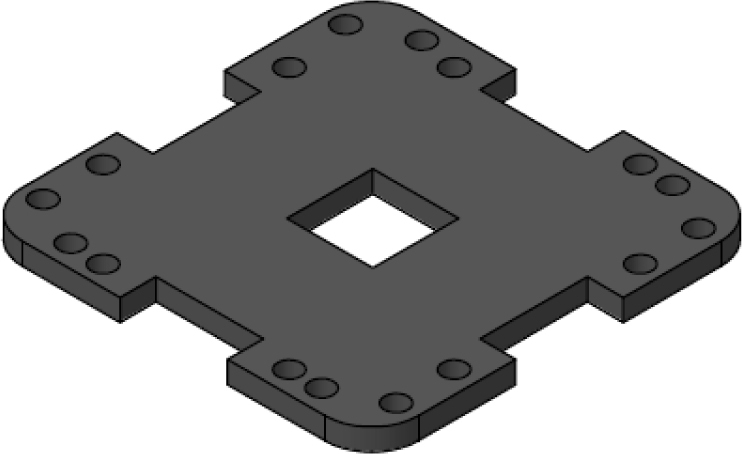
Top acrylic plate.

The electronic components of the system are mounted on three acrylic plates, each 3 mm thick. Part number 7, called top_acrylic_plate (Fig. 21), is a 55 mm × 55 mm plate with four holes for securing the ESP32 processor and four more for attaching the Mux TCA9548A. Additionally, it features eight auxiliary holes to connect this plate with the others. A 12 mm × 12 mm square cutout allows for cable routing, along with two 15 mm × 5 mm rectangular slots for the same purpose.

**Fig. 22:**
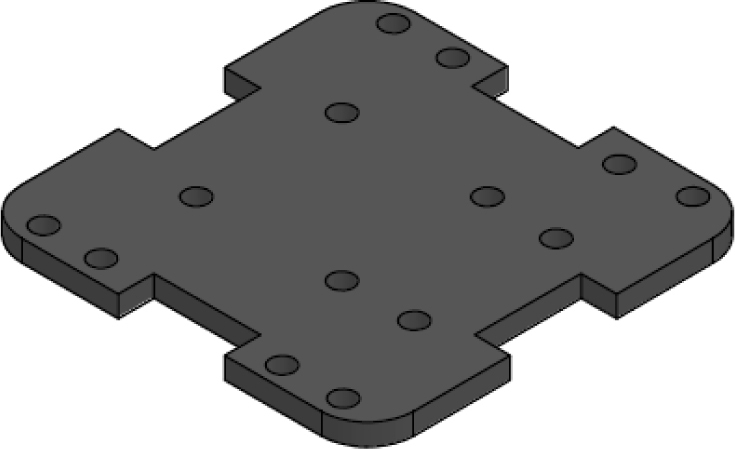
Middle acrylic plate.

**Fig. 23:**
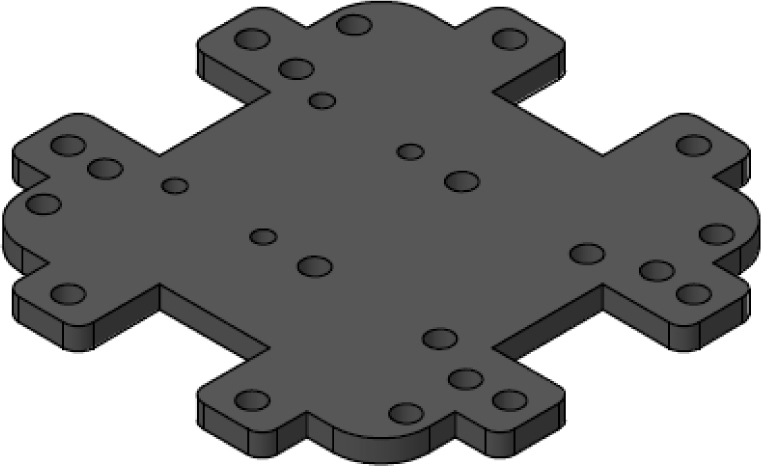
Bottom acrylic plate.

The middle_acrylic_plate (part number 8) is another 55 mm × 55 mm square plate. It has four holes to mount the OpenLog, two holes for attaching the SGP40 sensor, and also includes 15 mm × 5 mm slots for cable management, along with eight auxiliary holes for connecting to the other two plates, see Fig. 22. Part number 9, the bottom_acrylic_plate (Fig. 23), measures 67 mm × 67 mm and is used to mount the BME688 and SCD40 sensors. This plate includes eight holes for connecting it to the x_axis_support and z_axis_support, as well as another eight auxiliary holes for linking it to parts 7 and 8.

Although these parts were laser-cut, they can also be 3D printed. The designs are available in our repository here.

The FS3000 sensor, designed to measure wind speed, features a rectangular chip with a 6 mm × 2 mm opening. It is specifically built to detect the velocity of horizontal airflow passing through this cavity. In the current design, the sensor opening is positioned 8 mm from the flat face of the anemometer, ensuring that laminar winds enter without obstruction. Additionally, the 12 mm inlet window provides sufficient space for airflow to reach the 6 mm cavity.

However, the mechanical design of the anemometer, characterized by smooth faces and edges, introduces a critical limitation. It can generate turbulence, which affects measurement accuracy, particularly at wind speeds exceeding 3 m/s (as discussed in Section 8). In such cases, turbulent winds may not flow properly through the sensor slot, leading to inaccurate readings.

From a hardware perspective, the FS3000 is well-suited for detecting lower wind speeds due to its 125 ms response time, allowing it to take measurements up to eight times per second. This capability enables the sensor to capture moderate wind fluctuations, typical of low to moderate turbulence. However, in extreme turbulence or strong gusts, the sensor’s response time may be insufficient to track rapid variations accurately, thereby limiting its performance in such conditions.

## 4 Design files summary

The Table 3 summarizes the part names, license and file location; all files are available in STEP format.

**Table 3:** Summary of the design files used in the system development.

Name	Open source license	Location file
sensor_cover	GNU General Public License (GPL) 3.0	https://osf.io/ef96k
sensor_base	GNU General Public License (GPL) 3.0	https://osf.io/zj2ca
y_axis_support	GNU General Public License (GPL) 3.0	https://osf.io/gkbqc
x_axis_support	GNU General Public License (GPL) 3.0	https://osf.io/4jmp6
z_axis_SD_support	GNU General Public License (GPL) 3.0	https://osf.io/sfr49
z_axis_support	GNU General Public License (GPL) 3.0	https://osf.io/geupx
top_acrylic_plate	GNU General Public License (GPL) 3.0	https://osf.io/e9ypg
middle_acrylic_plate	GNU General Public License (GPL) 3.0	https://osf.io/bs4tv
bottom_acrylic_plate	GNU General Public License (GPL) 3.0	https://osf.io/7dtvk

## 5 Bill of materials

See Table 4.

**Table 4:** Components and specifications.

Designator	Component	Qty	Unit cost	Total cost	Source of materials
PLA filament	Structural	1	$10.79	$10.79	LINK
Acrylic	Structural	1	$9.98	$9.98	LINK
FS3000	Sensor	6	$59.95	$359.7	LINK
BME688	Sensor	1	$19.95	$19.95	LINK
SCD40	Sensor	1	$44.95	$44.95	LINK
SGP40	Sensor	1	$14.95	$14.95	LINK
TCA9548A	Multiplexor	1	$12.95	$12.95	LINK
SparkFun Thing Plus - ESP32	Processor	1	$22.50	$22.50	LINK
Sparkfun OpenLog	Data logger	1	$18.50	$18.50	LINK
SD memory extension cable	cable	1	$7.99	$7.99	LINK

## 6 Build instructions

**Fig. 24:**
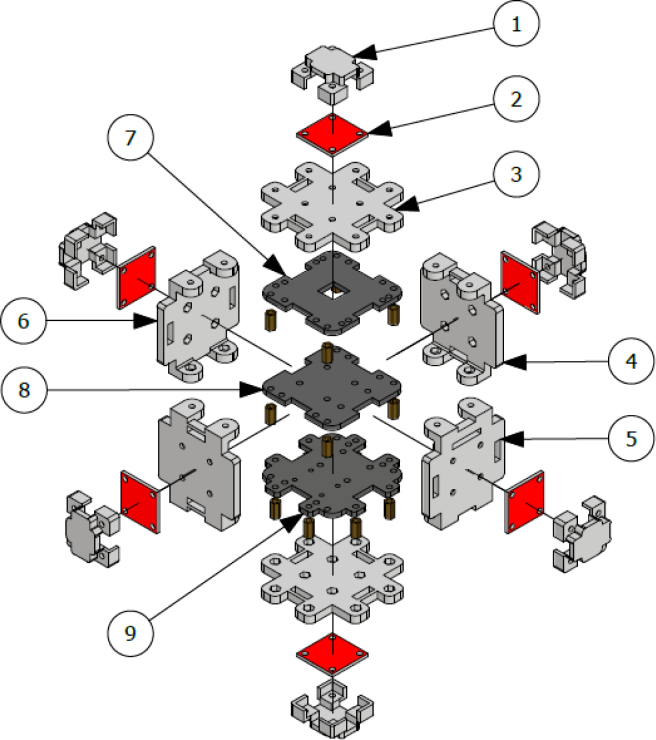
Assembly of the anemometer.

The assembly, shown in Fig. 24, resembles a cube, with a flow sensor mounted on each face, oriented toward the positive and negative axes of the Cartesian plane. All components are secured using M3 stainless steel screws and 10 mm high M-M metal spacer posts with internal threads. The assembly process is divided into three stages: first, the flow sensors are attached to each face of the anemometer; next, the internal acrylic parts are connected to the sensors; and finally, all components are assembled together.

In the first stage, parts 3, 4, 5, and 6 are assembled. These include the upper and lower y_axis_support, the left and right x_axis_support, the z_axis_SD_support and the z_axis_support. The sensor_base (part 2) is fixed to these supports, and the FS3000 flow sensor is mounted on top of it. Finally, the sensor_cover (part 1) is placed over the sensor. All these components are secured using four screws, with nuts embedded in the back of the main faces.

It is important to carefully consider the orientation of each sensor’s airflow measurement. The sensor measuring along the positive x-axis is oriented from west to east, while the sensor for the negative x-axis is positioned from east to west. The sensor for the positive y-axis is aligned from south to north, and the negative y-axis sensor is placed from north to south. For the z-axis, the positive sensor is positioned facing downward (capturing airflow from bottom to top), while the negative z-axis sensor faces upward (capturing airflow from top to bottom). The sensor cables pass through the rectangular slots. At the end of this first stage, the six faces of the anemometer are assembled but not yet connected to each other.

In the second stage of the assembly, the central components are joined together. Assembly begins with part 7 (top_acrylic_plate), where the ESP32 processor and the TCA9548A multiplexer are mounted. Four metal posts are attached to the outer corners of this board, which will be used to connect it to part 8 (middle_acrylic_plate). On part 8, the OpenLog data logger and the SGP40 sensor are secured. Additionally, four more metal posts are fixed to this board, allowing it to connect to part 9 (bottom_acrylic_plate). The BME688 and SCD40 sensors are attached to part 9.

Finally, in the third stage of the assembly, eight metal posts are added to part 9 to connect the internal and external sections of the assembly as follows: the metal posts on part 9 fit into the internal flanges of the left and right x_axis_support pieces, as well as the z_axis_SD_support and z_axis_support pieces, thereby joining the internal assembly with the four faces of the anemometer. To complete the assembly, the upper and lower y_axis parts are used to secure the flanges to the four previously assembled faces, stabilizing the structure of the anemometer (see Table 5).

The assembly of this device is simple, as the use of I${}^{2}$C communication facilitates the connections. It is recommended to avoid the use of metal tools, such as wrenches or screwdrivers, during the assembly process, as improper contact with the sensors or the processing board could cause serious failures. To facilitate assembly, first attach the sensors to their respective acrylic plates. Next, join the plates together using spacer posts. Finally, attach the center piece by aligning the faces where the wind sensors will be located.

**Table 5:** Required of elements for the main assembly.

Number	Name	Quantity
1	sensor_cover	6
2	sensor_base	6
3	y_axis_support	2
4	x_axis_support	2
5	z_axis_SD_support	1
6	z_axis_support	1
7	top_acrylic_plate	1
8	middle_acrylic_plate	1
9	bottom_acrylic_plate	1

**Fig. 25:**
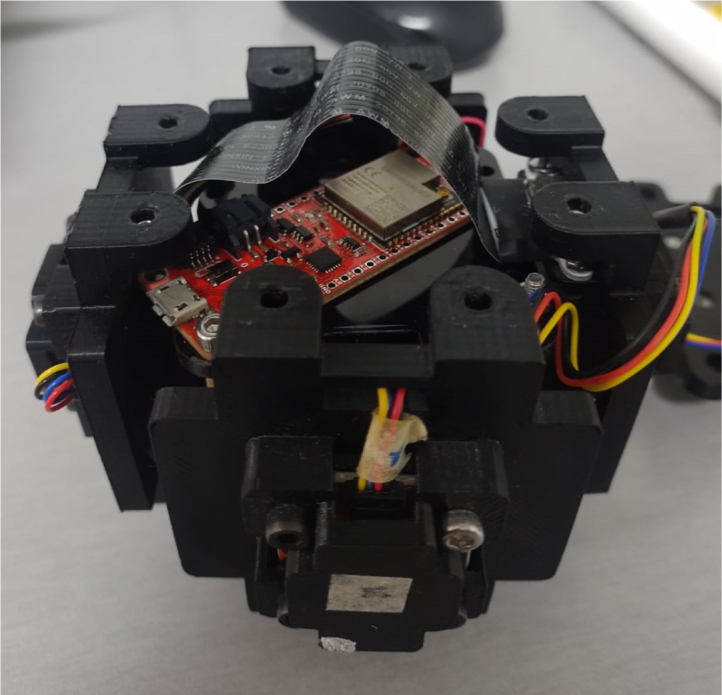
Assembly of the ESP32.

This compact design has limitations with cables that are too long, so it is important to select I${}^{2}$C cables of adequate size. A potential hazard, illustrated in Fig. 25, is that the microSD memory extension ribbon runs over the ESP32 card. Be careful when handling this ribbon to avoid breaking it. It is recommended to use an ESP32 without external soldered pins, as these could damage the ribbon. Also, there is no internal power supply included with the anemometer, so power is supplied through the USB port, via a cable coming out of one corner of the device.

**Fig. 26:**
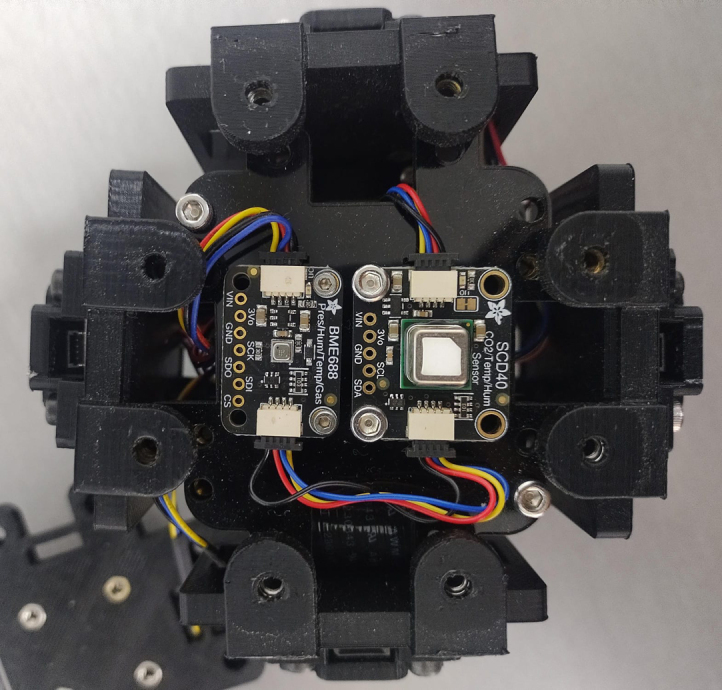
Sensors assembly.

## 7 Operation instructions

The device is easy to operate. Start by downloading the software from our repository, then open the code in the Arduino environment. Modify only the SSID and password to connect to a stable Wi-Fi network, and upload the code to the board. Ensure that the micro SD card is empty before inserting it into the device and powering it on. Place the device in the desired location for measurements and turn it on. When ready to view the data, power off the device before removing the micro SD card.

### 7.1 Troubleshooting and maintenance

If the device does not send data to the cloud and does not store the information in the micro SD memory it could be a disconnection of the sensors. The device code includes an operating detection phase for each sensor. To check its correct operation, connect the device to the computer and open the serial monitor to read the error messages. Data transfer is performed at 115 200 baud, and the device will generate error messages if there are problems with the WiFi connection, the connection to Ubidots or the connection of the sensors.

If an error message appears, check the WiFi connection or reboot the device if necessary. Then check the sensor connections one by one, making sure the LEDs are lit. If the error persists, it could be a power supply problem. In this case, check the battery status and measure the power consumption of the anemometer with a multimeter, this should not exceed 600 mA.

If the problem is related to data storage on the microSD memory, turn off the device, remove the memory, format and reinsert it. If the error persists, it could be due to an update in the OpenLog firmware, or again to power problems. At high current peaks, the device shuts down for safety, which may prevent proper data storage. The sensor electronics are exposed, as can be seen in Fig. 26 so care should be taken when using screws that exceed 1 cm in length, as they could make contact with the sensor electronics. It is also recommended to ensure that the nuts are tight, as loose nuts can affect the operation of the sensors. It is advisable to use lock nuts. In addition, be sure to install the device in an area where there is no risk of water entering the system.

This device is not designed to withstand mechanical loads or stresses. In case you need to reprint or replace any parts, always remember to disconnect the power supply before contacting the sensors. To prolong the life of this device, it is recommended to use it exclusively in indoor environments, avoiding exposure to direct sunlight. PLA, the main material of this device, degrades rapidly when exposed to extreme conditions, such as intense light or high humidity levels. Alternatively, to improve resistance over time, parts can be printed with more durable materials, such as ASA, nylon or polycarbonate. In this case, it will only be necessary to adjust the parameters of the 3D printer, without the need to modify the design of the parts.

## 8 Validation and characterization

The device was subjected to a test phase with the objective of verifying that the variables measured by the sensors are consistent with variations in environmental conditions, and that the flow sensors respond correctly to changes in wind speed and direction. The experiment was conducted in three phases. The first phase was carried out in a real environment to analyze the variations of environmental variables throughout the day in an enclosed space. For this phase, the BME688, SCD40 and SGP40 sensors were used, the anemometer was installed in a closed room in the Control Systems and Robotics Laboratory of the Instituto Tecnológico Metropolitano of Medellin, at an altitude of 1495 m. The space remained closed for four consecutive days without the presence of people, without ventilation or active air conditioning systems. During this time, data were collected and plotted to detect patterns.

The second phase focused on validating that the wind flow sensors adequately detect changes in wind speed and direction. For this, a closed metallic chamber of 40 cm × 40 cm × 40 cm was used, where the device was placed in the center. A fan acted as the wind source, and measurements were taken at different speeds and in different directions of the horizontal and vertical axis. Finally, tests are conducted by varying the wind direction at different speeds, and the measurements are compared against a commercial ultrasonic anemometer.

### 8.1 Assembly and manufacturing

**Fig. 27:**
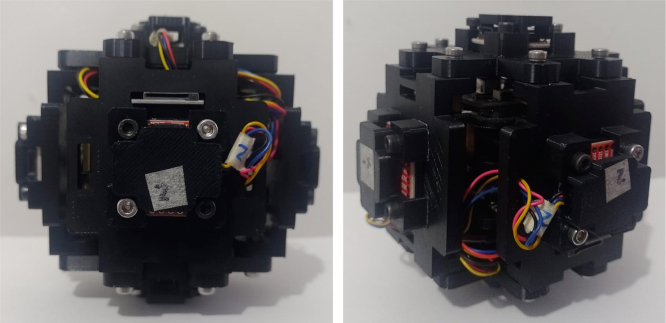
Final anemometer.

The assembly of this anemometer was done according to the instructions outlined in Section 6. Fig. 27 shows the final result of the 3D printed anemometer assembly. This device, easy to manufacture and compact, is ideal for indoor use. The small size (104 mm × 104 mm × 100 mm) facilitates installation and transportation, making it a practical and versatile option.

### 8.2 BME688 sensor testing

The BME688 sensor provides measurements of temperature, pressure, and humidity. This sensor was added to monitor the environmental conditions of the space. For temperature, this device measures within a range from −40 ${}^{\circ }C$ to 85 ${}^{\circ }C$. The average ambient temperature in Medellín during July, when the tests were conducted, was around 26 ${}^{\circ }C$. Since the tests were performed in an enclosed space without ventilation, it was expected that the measured temperature would be higher than average.

**Fig. 28:**
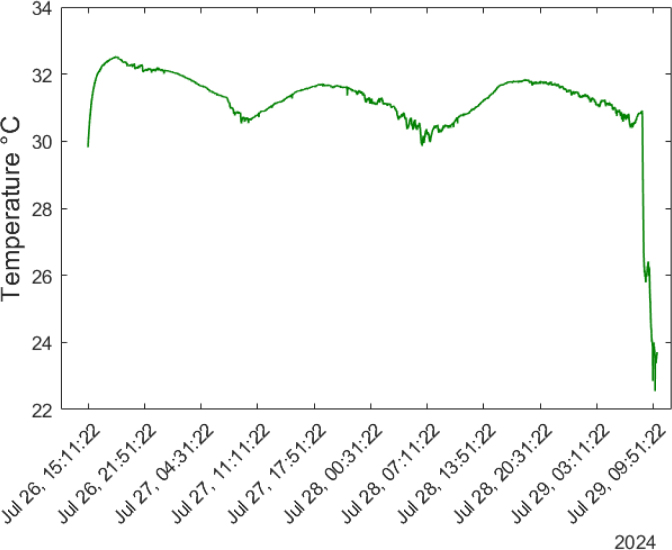
Temperature measured for four days in an enclosed space.

Fig. 28 shows the temperature behavior from July 26 to 29. The first data collected by the device was at 15:00, recording a temperature of 29 ${}^{\circ }C$. The graph shows how the temperature rises to 32.4 ${}^{\circ }C$ as night approaches, reaching its maximum between 15:00 and 17:00. The temperature then gradually drops to 30 ${}^{\circ }C$ in the morning hours, between 07:00 and 10:00. This cycle repeats over the three remaining days, with temperatures increasing at night and decreasing in the early morning. On the fourth day, corresponding to July 29, the laboratory was opened again, and the air conditioning was turned on. As a result, there was a sharp drop in temperature from 30 ${}^{\circ }C$ to 22 ${}^{\circ }C$ over a period of approximately two hours.

**Fig. 29:**
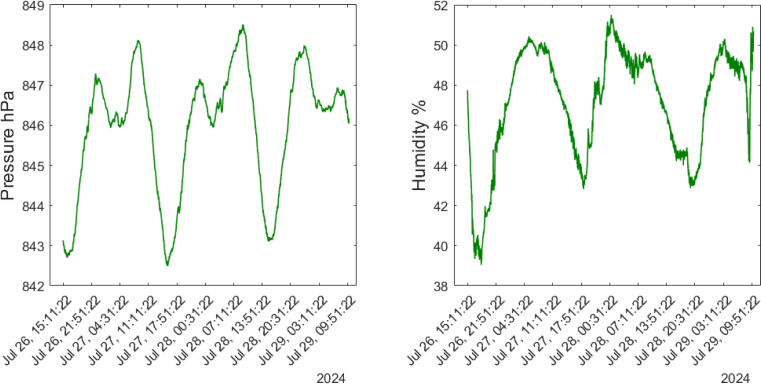
Pressure and humidity detected for four days in an enclosed space.

This device measures barometric pressure ranging from 300 hPa to 1100 hPa and humidity in percentages between 0% and 100%. Over the four-day analysis (Fig. 29), it can be observed that the pressure follows a consistent pattern, decreasing to around 822 hPa between 15:00 and 16:00. During the evening and early morning hours, two pressure peaks are detected: the first reaching 847 hPa around 23:00, and a second peak of 848 hPa between 08:00 and 09:00. Humidity exhibits an inverse relationship to temperature, decreasing when the temperature rises, and increasing as the temperature falls. On the first test night, July 26, humidity reached its minimum value of 39%, then increased to between 50% and 51% during the morning hours, from 04:00 to 10:00, and decreased again to 42% between 16:00 and 17:00. The periodic behavior of humidity closely mirrors that of temperature.

### 8.3 SCD40 sensor testing

**Fig. 30:**
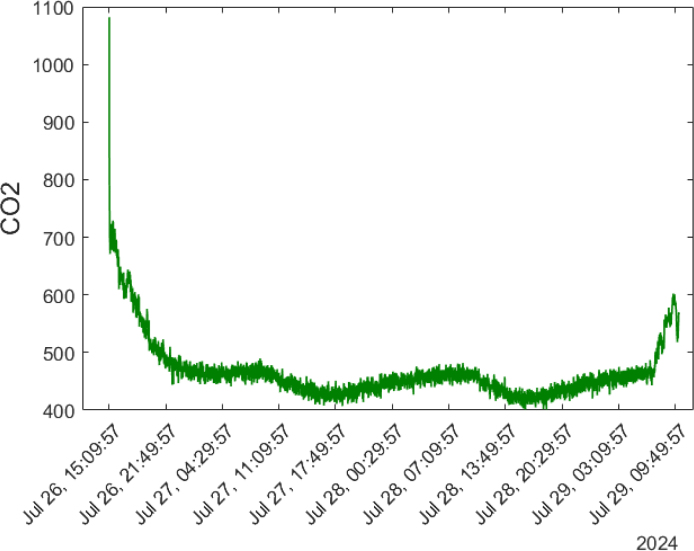
CO${}_{2}$ concentrations detected for four days in an enclosed space.

The SCD40 sensor also measures temperature and humidity, and provides CO${}_{2}$ measurements in the range of 400 to 2000 ppm. Fig. 30 shows the behavior of CO${}_{2}$ concentrations during the four days of testing. Upon startup, the device measures CO${}_{2}$ concentrations above 1000 ppm, but these readings stabilize after a few seconds. During the four days, CO${}_{2}$ concentrations remained between 400 ppm and 490 ppm. The highest concentrations were recorded in the morning hours between 06:00 and 10:00, peaking at 490 ppm, while the lowest concentrations were observed between 15:00 and 19:00. CO${}_{2}$ reached another maximum peak of 600 ppm on July 29 at around 09:00, just as the laboratory was opened to the public.

**Fig. 31:**
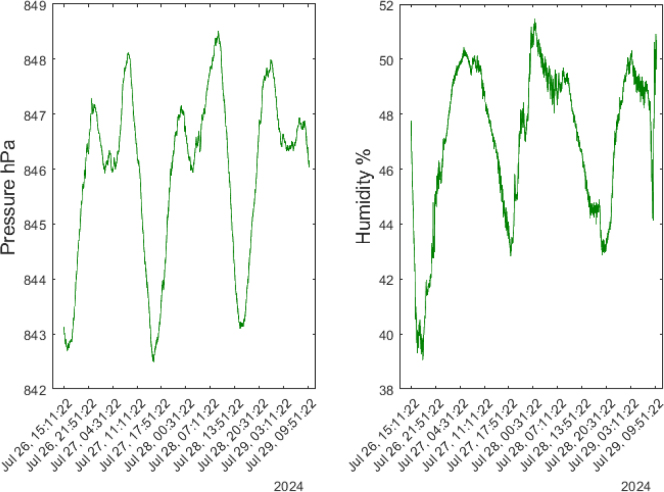
pressure and humidity detected on the SCD40 sensor.

The pressure and humidity measurements captured with the BME688 sensor were also recorded using the SCD40 sensor, as shown in Fig. 31. The lowest pressure values were observed in the afternoon, between 15:00 and 16:00, with a reading of 828 hPa. The maximum pressure was reached between 22:00 and 23:00, with a value of 847 hPa, and there was another peak between 8:00 and 9:00, reaching 848 hPa. For humidity measurements, a pattern similar to that of the BME688 sensor was observed, with humidity rising from 50% to 51% between 4:00 and 10:00, and decreasing to 43% between 16:00 and 17:00.

### 8.4 SGP40 sensor testing

**Fig. 32:**
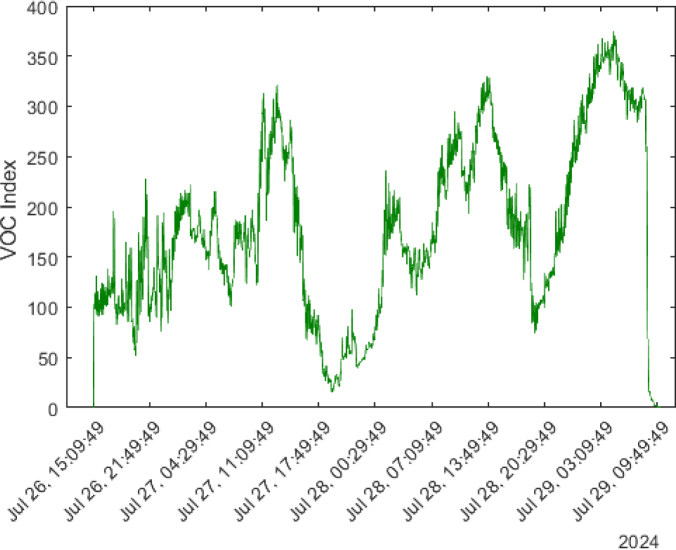
VOC index measured over four days in an enclosed space using the SGP40 sensor.

The SGP40 sensor provides air quality measurements by detecting the presence of VOC gases in the environment, delivering a VOC index ranging from 0 to 500. Upon activation, the device captures 100 data points during its stabilization phase. The detected VOC indices do not follow a specific pattern, as shown in Fig. 32.

During the first test day, the VOC indices averaged around 150. On the second test day, between 12:00 and 2:00 PM, the index increased to between 300 and 315. Similarly, on the third test day, the VOC index rose to between 320 and 330 at the same time. By the fourth day, the VOC indices further increased to 350 between 2:00 and 5:00 PM. These increases indicate that the longer the space remains enclosed, the greater the accumulation of VOCs.

However, at 8:30 AM, when the air conditioning was turned on, the VOC indices dropped drastically to a minimum of 7 within an hour. This sharp decrease in VOC levels suggests that indoor air quality significantly improves when ventilation sources, such as air conditioning, are introduced, promoting the circulation of gases and particulates in the environment.

### 8.5 FS3000 sensor testing

In this section, we focus on testing the performance of the anemometer under various wind conditions in terms of velocities and directions. The purpose of these tests is to evaluate the device’s capacity in detecting the direction and velocity of airflow in three dimensions.

For each measurement, we calculated the standard deviation to quantify the dispersion between the sensor’s readings and the average of all measurements. The formula used in this step is the standard deviation formula, as shown in Eq. (7), where: N is the number of data points in the sample, $x_{i}$ represents each individual value, and μ is the average value of the data.

(7) $$\sigma =\sqrt{\frac{1}{N}\overset{N}{\underset{i=1}{\sum }}{\left(x_{i}-\mu \right)}^{2}}$$

In this experiment, only tests with wind speeds of 1 m/s, 2 m/s and 3 m/s are reported. When evaluating the anemometer with higher speeds, it was observed that the measurements were limited to a maximum of 3.5 m/s, without correctly reflecting the increase in wind speed. This behavior suggests a limitation in the physical design of the device, which represents a challenge to be addressed in future research.

Initially, the focus was on testing the device’s ability to detect changes in speed. In this case, we concentrated on the resulting velocity calculated by the device, which is based on the independent velocities detected by the six flow sensors of the anemometer. The equations used are explained in Section 2.8.

**Fig. 33:**
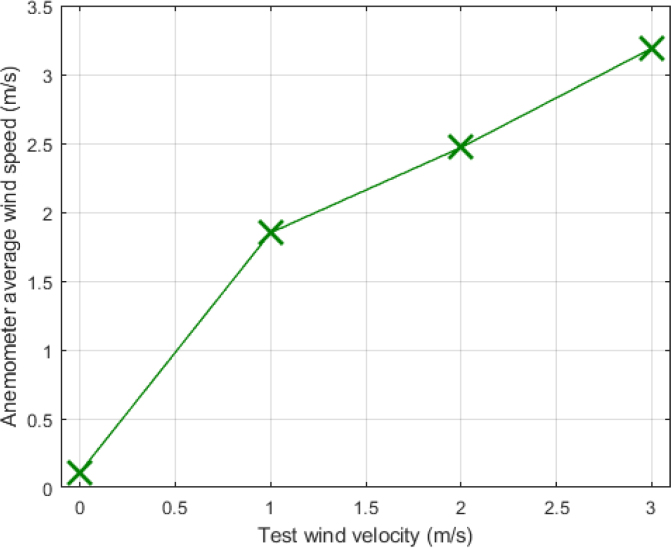
Wind speed captured by anemometer.

This test was conducted with wind speeds of approximately 0 m/s, 1 m/s, 2 m/s, and 3 m/s. For each speed, data were collected over a period of 5 min, and the average speeds captured by the device are shown in Fig. 33.

For the 0 m/s condition, when the wind source was off, the average velocity recorded by the device was 0.11 m/s with a standard deviation of 0.15 m/s. At 1 m/s, the average resultant velocity measured by the anemometer was 1.5 m/s with a standard deviation of 0.25 m/s. For the 2 m/s condition, the average resultant velocity captured was 2.47 m/s with a standard deviation of 0.22 m/s, and at 3 m/s, the average resultant velocity was 3.19 m/s with a standard deviation of 0.20 m/s.

These results demonstrate that the device effectively captures wind speed variations in indoor ventilation systems, providing correct measurements and data consistency. The integration of the six sensors in a cubic structure, along with the application of Eq. (4) to calculate the resultant velocity, proves to be an effective method for obtaining three-dimensional wind measurements.

**Fig. 34:**
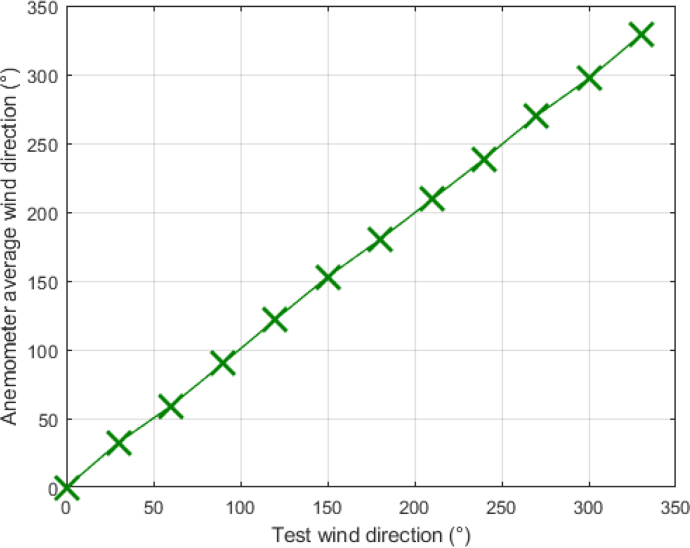
Wind horizontal direction captured by anemometer.

The detection of horizontal wind direction was tested at a fixed wind speed of 2 m/s. For the test, the anemometer was rotated every 5 min at the following positions: 0°, 30°, 60°, 90°, 120°, 150°, 180°, 210°, 240°, 270°, 300°, and 330°, see Fig. 34. Based on six readings from the flow sensors, the wind direction was determined according to Eq. (5).

Table 6 summarizes the test angles, the angles detected by the anemometer, and the standard deviation for each measurement. When the device was tested for the 0° direction, the wind flow was parallel to the measurement line of the positive × sensor, resulting in an average direction of 0° with a deviation of 0°. The same occurs when measuring winds along the negative x-axis, corresponding to the 180° direction, and with wind measurements along the positive and negative y-axes, corresponding to 90° and 270°, respectively.

As the anemometer is rotated, an increase in the deviation of the measurements is noted, as observed at the angles of 30°, 150°, 240° and 300°, where the deviations are 1.9°, 1.94°, 2.18° and 1.3°, respectively. The deviations increase to 3.7°, 3.15° and 3.14° for the 60°, 120°, 210° and 330° measurements. These measurement deviations are due to the mechanical configuration of the device and the arrangement of the sensors, since the corners of the device can cause bounces in the wind flow, thus altering the measurements. Since this device is intended for monitoring indoor wind and environmental conditions and for these applications high measurement accuracy is not required, it can be concluded that this device correctly detects the wind direction and provides consistent values in each test performed.

**Table 6:** Horizontal wind direction detection tests results.

Direction test	0°	30°	60°	90°	120°	150°
Direction measured	0°	32.6°	59°	90°	121.9°	152.9°
Standard deviation	0°	1.9°	3.7°	0°	3.3°	1.94°

Direction test	180°	210°	240°	270°	300°	330°

Direction measured	180°	209.2°	238.7°	270°	297°	328.8°
Standard deviation	0°	3.15°	2.18°	0°	1.3°	3.14°

**Fig. 35:**
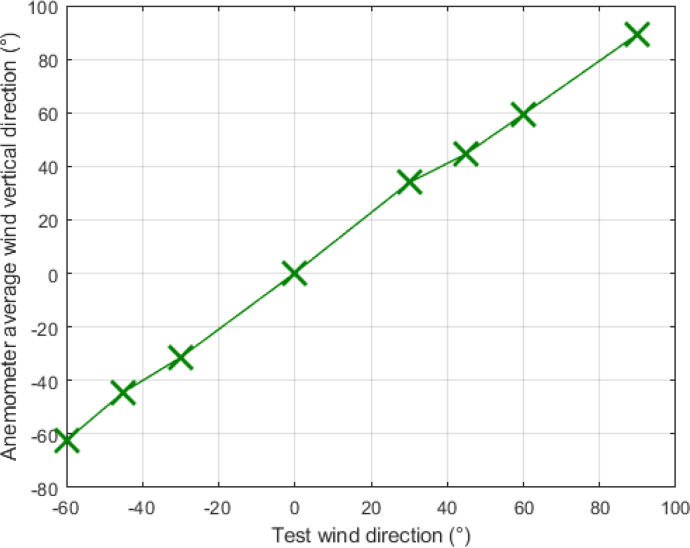
Wind vertical direction captured by anemometer.

The detection of vertical winds was tested using Eq. (6). These tests were performed at a constant speed of 2 m/s by rotating the anemometer in the following directions: 0°, 30°, 45°, 60°, 90°, −60°, −45°, and −30°, see Fig. 35. At 0°, the wind blows parallel to the z-axis; at 90°, it blows parallel to the positive x-axis; and at −90°, it blows parallel to the negative x-axis.

Table 7 summarizes the results obtained, showing the average vertical wind directions measured by the anemometer and the standard deviation for each case. In instances where the wind flow is parallel to any of the sensors — such as at 0° and 90° — the standard deviations are minimal, measuring 0.38° and 0.71°, respectively. As the wind direction varies, the standard deviation in the measurements increases, reaching maximum values of 3.02°, 2.68°, 2.84°, and 2.75° for vertical wind directions of 30°, 60°, −60°, and −30°, respectively.

The detection of the wind direction change could be made, demonstrating that the arrangement of six flow sensors and the proposed layout allow for effective measurement and monitoring of wind conditions in three dimensions.

**Fig. 36:**
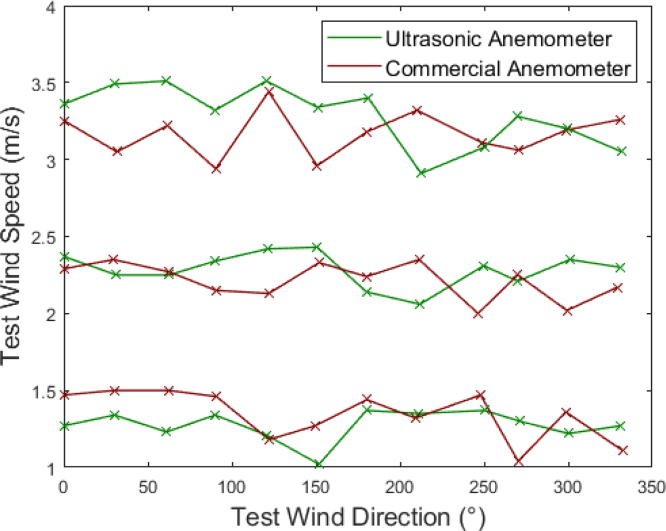
Comparison of the measured wind speed and wind direction between two anemometers.

The performance of the designed anemometer was evaluated by varying both wind speed and wind direction. For this purpose, the data obtained were compared with those recorded by the commercial Renke RS-CFSFX-*-3-EX ultrasonic anemometer, which measures wind speed and direction in two dimensions [50]. In this analysis, only the wind measurement in the horizontal plane was considered. The test consisted of exposing both anemometers to airflow with velocities of 1 m/s, 2 m/s and 3 m/s. For each of these velocities, the devices were rotated in 30° increments, from 0° to 330°. The objective of this experiment was to analyze the behavior of the developed anemometer under a constant wind speed at different wind directions. The Fig. 36 presents a performance comparison between the designed anemometer and the commercial reference anemometer. It illustrates the velocity and direction measured by both devices after rotation. The results indicate that both anemometers exhibit similar behavior in response to wind direction variations, validating the designed anemometer’s capability for wind direction and wind speed detection.

**Table 7:** Vertical wind direction detection tests results.

Direction	0°	30°	45°	60°	90°	−60°	−45°	−30°
Direction measured	0.14°	33.95°	44.7°	59.45°	89.42°	−62.4°	−44.37	−33.97°
Standard deviation	0.38°	3.02°	1.75°	2.68°	0.71°	2.84°	1.62°	2.75°

The absolute error formula is employed to quantify the discrepancies between the measurements obtained from the two anemometers. Table 8 summarizes these values for each evaluated wind speed and direction. The primary objective of this analysis is to assess the performance of the developed anemometer and verify its capability to accurately capture variations in wind speed and direction using commercial FS3000 sensors. Considering this objective, the device was not subjected to a prior calibration phase.

Both anemometers exhibit similar behavior in wind measurements. The maximum error in velocity was observed when the devices were tested with winds of 1 m/s and rotated 60°, resulting in a measurement offset of 0.57 m/s. For wind speeds of 2 m/s and 3 m/s, the maximum errors recorded were 0.33 m/s and 0.44 m/s, corresponding to wind directions of 300° and 30°, respectively. These results indicate that both devices demonstrate comparable performance, supporting the validity of the proposed anemometer in correctly measuring wind speeds.

**Table 8:** Detected angles and speed errors for different wind velocities.

Test	Wind velocity of 1 m/s		Wind velocity of 2 m/s		Wind velocity of 3 m/s	
angle (°)	Absolute direction error	Absolute speed error	Absolute direction error	Absolute speed error	Absolute direction error	Absolute speed error
0	0.1	0.2	0.2	0.08	0.6	0.11
30	0.11	0.46	1.8	0.1	1.49	0.44
60	1.27	0.57	0.1	0.02	0.78	0.29
90	0.82	0.12	0.53	0.19	0.42	0.38
120	1.51	0.03	0.38	0.29	1.41	0.07
150	2.36	0.25	1.1	0.1	0.09	0.38
180	0.18	0.07	0.01	0.1	0.79	0.22
210	2.03	0.03	0.56	0.29	2.41	0.41
240	2.41	0.1	3.38	0.31	1.28	0.03
270	0.89	0.26	0	0.04	0.66	0.22
300	1.49	0.14	1.87	0.33	1.4	0.01
330	1.08	0.16	1.42	0.13	0.91	0.21

Larger variations were observed in the wind direction measurements. For winds of 1 m/s, the greatest deviations were recorded at 150°, 210°, and 240°, with lags of 2.36°, 2.03°, and 2.14°, respectively. At a wind speed of 2 m/s, the largest offset was 3.38° at 240°, while for winds of 3 m/s, the maximum deviation was 2.14° at 210°. In contrast, the remaining wind direction measurements across different speeds exhibited minimal offsets. These findings suggest that the developed device effectively tracks wind direction, despite some variations in specific cases. However, these deviations are not considered significant, as the intended application of the device does not require highly precise measurements.

### 8.6 Power consumption

**Fig. 37:**
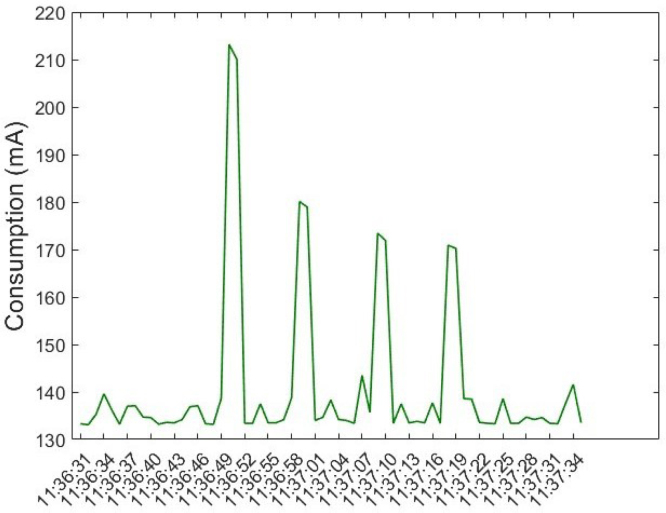
Consumption of the anemometer during data transmission.

To evaluate the impact on energy consumption, measurements were made of the current consumed by the anemometer during the sending of four packets of information. The Fig. 37 shows the current consumption peaks that occur each time a packet is sent to the cloud. The minimum consumption of the device is, on average, 133 mA, while the peaks vary between 213 mA and 170 mA. Although this consumption is within the limits of the ESP32, which supports a maximum consumption between 500 mA and 600 mA, the integration of additional sensors is not recommended to avoid overloading the device. In addition, when connecting an external power supply, it is necessary to verify that it provides adequate current. When the device is configured to send data in real time, the power consumption increases. For this reason, timeouts were implemented between measurements from each sensor. After capturing data from each sensor (FS3000, BME688, SCD40, SGP40), a timeout of 1 s is set before proceeding with the next measurement. Once a data packet is sent to Ubidots, a 5 s timeout is implemented before proceeding with the next update.

It is important to note that this device is designed primarily for use in indoor environments, where real-time measurements are not strictly necessary. The update times of the variables can be extended even up to several minutes without affecting the efficiency of the application. As for the sensors, each has a different response time: The FS3000 has a response time of 125 ms, the BME688 is set to a response time of 500 ms, the SGP40 has a response time of 10 s, and the SCD40 is the sensor with the longest response time, which can reach up to 120 s when measuring temperature, humidity and CO${}_{2}$. Since the sensor measurements are not required to be synchronized, the sampling rate between sensors was not adjusted. In addition, for each reading, the timestamp at which the measurement was captured is stored, allowing detailed analyses to be performed later, even if the measurements are not taken at regular intervals. A temperature compensation phase was not implemented, as the FS3000 sensor datasheet specifies an operating temperature range of −20 °C to ＋85 °C.

### 8.7 Discussion

The main limitations identified in this prototype are related to the mechanical configuration of the device. Although the results obtained have been satisfactory, achieving higher accuracy in specific applications will require a dedicated calibration phase using certified techniques and instruments. Furthermore, future studies should explore alternative mechanical configurations for sensor placement to enhance the system’s aerodynamic performance.

This device is designed to measure wind speeds directly impinging on the sensor input. However, further research is needed to assess its performance under turbulent flow conditions with varying characteristics. Such evaluations will facilitate adjustments to the prototype to improve the accuracy of turbulence measurements.

In future work, optimizing power consumption will be a key consideration. Potential solutions include integrating low-power sensors, designing energy-efficient algorithms, or exploring the possibility of powering the sensors from an external source to reduce battery dependency and extend operational longevity.

### 8.8 Conclusions

A three-dimensional anemometer with integrated environmental sensors for indoor air quality monitoring was developed and tested. The device employs six one-dimensional flow sensors to measure wind speed along the three positive and three negative axes of the Cartesian plane. All components were 3D-printed, making the device lightweight, easy to assemble, install, and transport.

The prototype, based on hot-wire sensors, overcomes the limitation of conventional single-direction wind measurements by enabling three-dimensional wind speed and direction characterization. The use of a six-sensor array enhances angular coverage, providing a more comprehensive representation of wind dynamics compared to traditional anemometers. This capability is particularly relevant for indoor airflow monitoring applications.

Additionally, the integration of sensors for temperature, humidity, CO${}_{2}$, and volatile organic compounds (VOCs) enables a detailed assessment of indoor climatic conditions and air quality. This feature supports health risk evaluations in enclosed spaces and informs timely interventions.

This study presents a novel, cost-effective, and reliable approach to three-dimensional wind measurement, combined with environmental monitoring, in a single, easy-to-manufacture device using commercially available components. The proposed system is suitable for various applications requiring indoor airflow characterization and air quality assessment.

## CRediT authorship contribution statement

**Elizabeth Ospina-Rojas:** Writing – review & editing, Writing – original draft, Validation, Investigation, Conceptualization. **Juan Botero-Valencia:** Writing – review & editing, Writing – original draft, Supervision, Project administration, Methodology, Conceptualization. **Daniel Betancur-Vasquez:** Writing – original draft, Validation, Methodology. **Joshua M. Pearce:** Writing – review & editing, Writing – original draft, Supervision, Methodology, Formal analysis, Conceptualization.

## Human and animal rights

No human or animal studies were conducted in this work.

## Declaration of competing interest

The authors declare that they have no known competing financial interests or personal relationships that could have appeared to in influence the work reported in this paper.
